# Hexokinase 3 enhances myeloid cell survival via non-glycolytic functions

**DOI:** 10.1038/s41419-022-04891-w

**Published:** 2022-05-11

**Authors:** Kristina Seiler, Magali Humbert, Petra Minder, Iris Mashimo, Anna M. Schläfli, Deborah Krauer, Elena A. Federzoni, Bich Vu, James J. Moresco, John R. Yates, Martin C. Sadowski, Ramin Radpour, Thomas Kaufmann, Jean-Emmanuel Sarry, Joern Dengjel, Mario P. Tschan, Bruce E. Torbett

**Affiliations:** 1 Division of Experimental Pathology, Institute of Pathology, University of Bern, Bern, Switzerland; 2 Graduate School of Cellular and Biomedical Sciences, University of Bern, Bern, Switzerland; 3 Department of Immunology and Microbiology, Scripps Research, La Jolla, CA USA; 4 Department of Biology, University of Fribourg, Fribourg, Switzerland; 5 Department of Molecular Medicine, Scripps Research, La Jolla, CA USA; 6 Department of Medical Oncology, Inselspital, Bern University Hospital, University of Bern, Bern, Switzerland; 7 Tumor Immunology, Department for BioMedical Research (DBMR), University of Bern, Bern, Switzerland; 8 Institute of Pharmacology, University of Bern, Bern, Switzerland; 9 Centre de Recherches en Cancérologie de Toulouse, Université de Toulouse, Inserm, CNRS, Toulouse, France; 10 Centre Hospitalier Universitaire de Toulouse, Toulouse, France; 11 Department of Pediatrics, School of Medicine, University of Washington, Seattle, WA USA; 12 Center for Immunity and Immunotherapies, Seattle Children’s Research Institute, Seattle, WA USA; 13 Institute for Stem Cell and Regenerative Medicine, Seattle, WA USA

**Keywords:** Cell biology, Cancer

## Abstract

The family of hexokinases (HKs) catalyzes the first step of glycolysis, the ATP-dependent phosphorylation of glucose to glucose-6-phosphate. While HK1 and HK2 are ubiquitously expressed, the less well-studied HK3 is primarily expressed in hematopoietic cells and tissues and is highly upregulated during terminal differentiation of some acute myeloid leukemia (AML) cell line models. Here we show that expression of HK3 is predominantly originating from myeloid cells and that the upregulation of this glycolytic enzyme is not restricted to differentiation of leukemic cells but also occurs during ex vivo myeloid differentiation of healthy CD34^+^ hematopoietic stem and progenitor cells. Within the hematopoietic system, we show that HK3 is predominantly expressed in cells of myeloid origin. CRISPR/Cas9 mediated gene disruption revealed that loss of HK3 has no effect on glycolytic activity in AML cell lines while knocking out HK2 significantly reduced basal glycolysis and glycolytic capacity. Instead, loss of HK3 but not HK2 led to increased sensitivity to ATRA-induced cell death in AML cell lines. We found that *HK3* knockout (*HK3*-null) AML cells showed an accumulation of reactive oxygen species (ROS) as well as DNA damage during ATRA-induced differentiation. RNA sequencing analysis confirmed pathway enrichment for programmed cell death, oxidative stress, and DNA damage response in *HK3*-null AML cells. These signatures were confirmed in ATAC sequencing, showing that loss of HK3 leads to changes in chromatin configuration and increases the accessibility of genes involved in apoptosis and stress response. Through isoform-specific pulldowns, we furthermore identified a direct interaction between HK3 and the proapoptotic BCL-2 family member BIM, which has previously been shown to shorten myeloid life span. Our findings provide evidence that HK3 is dispensable for glycolytic activity in AML cells while promoting cell survival, possibly through direct interaction with the BH3-only protein BIM during ATRA-induced neutrophil differentiation.

## Introduction

The first and irreversible step of glycolysis, the ATP-dependent phosphorylation of glucose to glucose-6-phosphate (G6P), is catalyzed by hexokinases (HK). Four different isoforms have been identified in mammalian cells (*HK1*, *HK2*, *HK3*, and *HK4*, also known as glucokinase GCK) [1, 2]. HKs promote and sustain a concentration gradient that facilitates glucose entry and therefore ensure the initiation of glucose-dependent pathways [3–5]. HK1 and 2 are expressed in various tissues [6], HK4, or GCK, has distinct biochemical properties and is half the size of the other hexokinases (reviewed in [7]). It is primarily known as a glucose sensor in pancreatic and liver tissue [8, 9]. Due to its distinction from HK1–3 it was excluded from this present study. HK2 is highly expressed in embryonic tissues [6] and has also been implicated in tumor growth and metastasis with cancer cells generally expressing high levels of HK2 and low levels of HK1 [10–13]. In addition, HK2 is involved in lung and breast cancer initiation and maintenance [12]. Our group previously identified HK3 as a transcriptional target of the hematopoietic transcription factor PU.1 (SPI1) during acute promyelocytic leukemia (APL) differentiation [14]. HK3 is distinguished from isoforms 1 and 2 by the absence of a 21 amino acid sequence in the N-terminal domain that enables HK1 and HK2 to bind to the outer mitochondrial membrane [15, 16]. HK1 and HK2 were reported to inhibit mitochondrial sensitization to apoptosis by restricting access of the proapoptotic BCL-2 protein Bax to the mitochondria [17]. A cytoprotective role for HK1 and HK2 was further highlighted by the enhanced sensitivity to drugs in combination with their inhibition [14, 18–21]. HK3 lacks the mitochondrial binding domain, has been detected mainly in the soluble fraction of the cytoplasm and is associated with perinuclear localization [22]. Other unique features of HK3 include substrate inhibition at higher, but still physiological levels of glucose, which in turn is antagonized by ATP [6]. Apart from this, very little is known about the function of HK3.

## Material and methods

### Compounds

Miltirone (CFN98531, AOBIOUS, CAS 27210-57-7), ST1926 (SML2061, Sigma-Aldrich, CAS 496868-77-0), 2-DG (31060, Sigma-Aldrich, CAS 154-17-6), CB-839 (533717, Sigma-Aldrich, CAS 1439399-58-2), Etomoxir (236020, Sigma-Aldrich, CAS 828934-41-4), and *N*-acetyl-l-cysteine NAC (A9165, Sigma-Aldrich, CAS 616-91-1) were used at indicated concentrations and duration.

### Primary cells, cell lines, and culture conditions

Human cord blood was obtained from the Cleveland Cord Blood Center. Under approved institutional protocol and in accordance with the Declaration of Helsinki, CD34^+^ cells were isolated using either the EasySep Human Cord Blood CD34 Positive Selection Kit according to the manufacturer’s instructions (STEMCELL Technologies) or the human CD34 MicroBead Ultrapure kit (Miltenyi Biotech) followed by automatic separation on the autoMACS Pro Separator (Miltenyi Biotech). For PBMC isolation, whole blood was obtained from the Scripps Normal Blood donor Service. PBMCs were isolated over a Ficoll-density gradient and CD33^+^ or CD4^+^ cells were purified using magnetic bead separating with the respective antibodies (CD33 MicroBeads, Miltenyi Biotech/CD4 EasySep isolation kit, Stemcell Technologies). Activation of T cells was induced by the addition of CD3/CD28 Dynabeads (Thermo Fisher) at a bead to cell ratio of 1:1.

The human AML cell lines NB4 and HL60 were maintained in RPMI-1640 with 10% fetal bovine serum (FBS) and 1% l-glutamine in a 5% CO_2_–95% air humidified atmosphere at 37 °C. Myeloid differentiation of NB4 and HL60 cells were induced with either 1 μM of ATRA (dissolved in DMSO) over a course of 4–6 days for neutrophil-like differentiation or 1α,25-dihydroxy vitamin D_3_ (VitD3) for 3 days for differentiation towards a monocyte-macrophage-like lineage.

HEK 293 T cells were maintained in DMEM (Sigma-Aldrich, St. Louis, MO, USA), supplemented with 5% FBS, 1% penicillin/streptomycin, 1% Hepes (Sigma-Aldrich), and were kept in 7.5% CO_2_–95% air humidified atmosphere at 37 °C.

### Real-time quantitative reverse transcription-PCR (RT-qPCR)

Total RNA was extracted using the RNeasy Mini Plus RNA isolation kit according to the manufacturer’s protocol (Qiagen, Hombrechtikon, Switzerland). Total RNA was reverse transcribed using All-in-one cDNA Synthesis SuperMix (Bimake, Munich, Germany). qPCR was performed using either the ABI PRISM^®^ 7700 Sequence Detection System (Applied Biosystems, Rotkreuz, Switzerland) or a LightCycler 480 Instrument (Roche, Basel, Switzerland). For quantification of *HK1–3* mRNA the Taqman^®^ Gene Expression Assays *HK1* Hs00175976_m1-44553320, *HK2* Hs00606086_m1-44553320, *HK3* Hs01092839_m1 (Applied Biosystems) were used. *HMBS* and *ABL* reference gene primers and probes have been described earlier [23, 24]. N-fold changes were calculated using the ^∆∆^Ct method of relative quantification.

### Cell lysate preparation and western blotting

Whole-cell extracts were prepared using 8 M UREA 0.5% Triton-X lysis buffer and 10–75 μg total protein was loaded onto denaturing polyacrylamide gels (Bio-Rad). Blots were incubated with the primary antibodies in TBS 0.05% Tween-20/ 5% milk or 5% BSA overnight at 4 °C, incubated with HRP coupled secondary goat anti-rabbit and goat anti-mouse antibody (Cell signaling) at 1:5,000 for 1 h at room temperature. Primary antibodies used were anti-HK1 (Cell Signaling, #2024, 1:1000), anti-HK2 (Cell signaling, #2867, 1:1000), anti-HK3 (Thermo Fisher Scientific, #PA5-29304, 1:500), anti-yH2AX Ser139 (Cell Signaling, #2577, 1:1000), anti-cleaved Caspase-3 (Cell Signaling, #966, 1:1000 Milk/TBS-T), anti-Puma (Cell Signaling, #12450, 1:1000 BSA/TBS-T), anti-Noxa (Enzo Life Sciences, ALX-804-408-C100, 1:1000 Milk/TBS-T), anti-BCL-2 (Santa Cruz, sc-509, 1:1000 BSA/TBS-T) and anti-BIM (Cell Signaling, #2933, 1:1000 BSA/TBS-T). Cytoplasmic and nuclear fractionation was performed as described before [25]. Uncropped western blots are shown in the supplement.

### Lentiviral vectors and CRISPRv2 mediated knockout

Full-length HK3 as well as a D542A mutant HK3 were cloned into a pFG12 expression vector (kind gift from David Baltimore, Addgene plasmid # 14884 [26]). The constructs express eGFP and the hexokinase sequence, bridged by a P2A self-cleaving peptide sequence for equimolar expression levels without the generation of a fusion protein.

Hexokinase knockout AML cell lines were generated using the lentiCRISPRv2 vector containing the Cas9 endonuclease gene and a puromycin selection marker (a gift from Feng Zhang; Addgene plasmid # 52961 [27]). Lentiviral vectors for CRISPR knockouts were generated by transient transfection of the corresponding plasmid and third-generation packaging plasmids pMD2.G (VSV-G), pMDLg/pRRE (gag and pol), and pRSV-Rev (rev) into HEK 293 T cells. Briefly, cells were transfected using calcium phosphate, and the supernatant was collected 72 h post-transfection. The media was filtered through a 0.45μm nitrocellulose membrane and concentrated over a 20% sucrose gradient by ultracentrifugation at 19,400 rpm for 2 h and 20 min at 4 °C in a Beckman SW28 rotor and resuspended in RPMI-1640. About 1.25 × 10^5^ NB4 or HL60 cells were incubated with 2–50 μl of concentrated virus and 8 μg/ml polybrene for 30 min in a humidified incubator at 37 °C, then centrifuged at 1000 × *g* for 90 min at 37 °C and incubated overnight. Two to seven days post spinoculation cells were selected with 1.5 µg/ml puromycin for 4 days followed by 3 days of 0.5 µg/ml. Following selection, single clone populations were grown by limiting dilution assay in a 96-well format in PRMI-1640 supplemented with 20% FBS.

To confirm knockouts, genomic DNA was isolated using Qiagen’s DNeasy Blood&Tissue kit according to the manufacturer’s instructions and genomic regions around the CRISPR guide RNA binding sites were amplified. Subsequent Sanger Sequencing confirmed INDELs at the Cas9 cut site.

### Transduction, expansion, and differentiation of primary CD34^+^ cells

Isolated CD34^+^ cells were expanded for 5–8 days in IMDM supplemented with 10% FBS, 100 ng/ml SCF, 50 ng/ml IL-3, 50 ng/ml IL-6, and 100U/ml penicillin and 100 µg/ml streptomycin (P/S) and differentiated towards neutrophils in RPMI-1640 medium with 50 ng/µl G-CSF, 5 ng/µl IL-6, 10% FBS, and 1% P/S over a course of 9–12 days. For monocytic/macrophage differentiation, CD34^+^ cells were differentiated in RPMI-1640 medium with 50 ng/ml GM-CSF, 20 ng/ml M-CSF, 10% FBS, and 1% P/S. After 3 days of differentiation, GM-CSF was removed from the medium and cells were differentiated for an additional 6–9 days. Cell density was closely monitored and maintained at 0.5–1 × 10^6^ cells/ml over the duration of differentiation. For lentiviral transduction, cells were transduced as described previously [28].

### Generation and analysis of CRISPR/Cas9 mediated HiBiT tagging of endogenous HK2 and HK3

For the generation of HiBiT-tagged lines, the protocols described [29, 30] were followed. Recombinant eGFP-Cas9 protein was purchased from Sigma (ECas9GFPPR). sgRNA, ssODN, and HDR enhancer were purchased from Integrated DNA Technologies (IDT), sequences can be found in Supplementary Table S1. Briefly, a sgRNA:Cas9 ratio of 7.5:1 and a total amount of 10 pmol of Cas9 protein was used per electroporation. To form RNP complexes, Cas9 and sgRNA were incubated in buffer R (Neon electroporation kit, Thermo Fisher) for 15 min at room temperature. One microliter of 60 µM ssODN was added. Cells were washed in PBS and 50 K cells were resuspended in buffer R. RNP/ssODN solution was added and cells were electroporated with one pulse at 1350 V and a pulse width of 35 ms using a 10 µL tip supplied with the Neon electroporation kit. After a 96 h recovery period, a limiting dilution assay was performed and emerging monoclonal populations were screened for integration of the HiBiT-tag using lytic detection as well as PCR amplification of the insert. Luminescent detection of HiBiT was performed using Nano-Glo HiBiT Lytic Detection System as well as Nano-Glo HiBiT Blotting System according to the manufacturer’s protocol (Promega). The signal was detected on a Tecan Infinite 2000 plate reader using an integration time of 1000 ms for the lytic detection assay (Tecan Group, Männedorf, Switzerland) or alternatively on a ChemiDoc XRS + Imaging System for blotted protein analysis (Bio-Rad, Basel, Switzerland).

### Proximity ligation assay

Proximity ligation assay was performed using Duolink^®^ in situ PLA (DUO92101, Sigma-Aldrich). After treatment, cells were incubated for 2 h on poly-l-lysine coated eight-well chamber slides (#94.6170.802, Sarstedt, Nümbrecht, Germany). Cells were fixed for 10 min in 2% PFA and permeabilized for 5 min in PBS/0.1% Triton-X100. Cells were blocked in PBS/10% BSA for 1 h at RT. Immunostaining with primary antibodies (anti-HK3 Thermo Fisher #PA5-29304, anti-HiBiT Promega (clone 30E5), anti-BIM Cell Signaling #2933, anti-VDAC Cell Signaling #4661) at a concentration of 1:100 for 1 h at 37 °C. To perform the PLA, the manufacturer’s protocol was followed. Data acquisition were performed on an Olympus IX81 FV1000 confocal microscope and analyzed using ImageJ software.

### HiBiT and BIM pulldown

HiBiT-tagged proteins were pulled down using the HiBiT pulldown kit containing Halo-tagged LgBiT protein, DrkBiT Peptide, and Magne^®^ HaloTag^®^ Beads (CS1967B02, Promega) according to the manufacturer’s protocol. HL60 cells expressing endogenous HK3-HiBiT were compared against cells expressing HK2-HiBiT as well as a no-HiBiT expressing control. Samples were treated with ±1 µM ATRA for 3 days. Protein was isolated using Mammalian Lysis Buffer (G9381, Promega) supplemented with a proteinase inhibitor cocktail (Roche). A total amount of 500–1000 ug of protein was incubated with Halo-tag^®^-LgBiT protein and incubated for complexation. Beads were added and the suspension was incubated at room temperature for 3 h. After washing, the bead-bound protein was eluted using DrkBiT Peptide and flash-frozen.

For endogenous BIM immunoprecipitation (IP) and HK3 co-IP the following kit was used: Pierce™ Immunoprecipitation, Crosslink Magnetic IP/Co-IP Kit (Thermo Scientific, # 88805). Briefly, 0.88 µg BIM antibody (anti-BIM (Cell Signaling, #2933) were cross-linked to Protein A/G magnetic beads before incubation with 1 mg of protein lysate for 1.5 h at RT on an end-to-end rotator. Thereafter, flow-through was saved and beads were washed with TBS followed by water. Elution was done according to instructions. Input, flow-through, and IP were loaded on a western blot gel for detection of HK3, BIM, and BCL-2.

### Hexokinase activity assay

Total hexokinase activity was assessed using the hexokinase activity colorimetric assay (ab136957, Abcam, USA). Hexokinase activity of 2.5 × 10^5^ cells per well was measured according to the manufacturer’s instructions. Absorbance readings were measured after an incubation time of 40 min.

### Oxygen consumption and extracellular acidification rate measurements

Metabolic profiling was done using a Seahorse XF96 flux analyzer (Agilent, Santa Clara). Mitochondrial respiration was assessed with the Agilent Seahorse XF Cell Mito Stress Test Kit. For investigation of glycolytic activity, the Agilent Seahorse XF Glycolytic Rate Assay Kit was used. Experiments were conducted following the manufacturer’s instructions for non-adherent cells using Cell-Tak extracellular matrix protein preparation to prepare adherent monolayer cultures of NB4 and HL60 cell lines, including knockdown and knockout sublines. For both assays, 1.6 × 10^5^ cells were seeded in a Seahorse XF96 cell culture plate pre-coated with Cell-Tak. Mitochondrial respiration was measured at baseline and following sequential injections of ATP-synthase inhibitor oligomycin, carbonyl cyanide-p-trifluoromethoxyphenylhydrazone (FCCP) to stimulate maximal oxygen consumption by uncoupling proton transport across the mitochondrial membrane and finally rotenone/antimycin A, inhibitors of of complex I and III, respectively. The glycolytic activity was measured at baseline and following sequential injections of rotenone/antimycin A to inhibit mitochondrial respiration and 2-deoxy-d-glucose (2-DG) to disrupt the glycolytic pathway.

### Caspase-Glo^®^ 3/7 assay, pan-caspase inhibition, alamarBlue^®^ and CellTiter-Glo^®^

The caspase-3/-7 activity was measured using the Caspase-Glo^®^ 3/7 assay according to the manufacturer’s instructions (#G8090, Promega AG). Cells were treated in a six-well format. At each timepoint of the assay, 7.5 × 10^4^ cells/well were transferred to a 384-well plate and assessed for their Caspase-3/-7 activity.

Caspase activity was inhibited using pan-caspase inhibitor Q-VD-OPh (#S7311, Selleckchem/Lubio Science, Zurich, Switzerland) at indicated concentrations. Q-VD-OPh was added at the beginning of the experiment and was replenished once after 48 h.

To assess relative viability upon ATRA treatment, the alamarBlue^®^ assay was used (#DAL1025, Thermo Fisher). Cells were cultured in 96-well plates for the duration of the assay and 10% alamarBlue^®^ was added at the end. Absorption was measured after 3 h.

When measuring viability upon nutrient restriction, CellTiter-Glo^®^ luminescent cell viability assay (#G7570, Promega AG) was used to assess viability dependent on ATP availability. The assay was performed according to the manufacturer’s protocol in a 384-well format using 12.5 × 10^4^ cells/well.

### NBT

About 5 × 10^5^ cells were resuspended in a 0.2% nitroblue tetrazolium (NBT) solution containing 40 ng/ml PMA and incubated for 15 min at 37°. Cells were then washed with PBS and subjected to cytospin. Counterstaining was done with 0.5% Safranin O for 5 min (HT90432; Sigma-Aldrich, Switzerland). The NBT positive and negative cells were photographed under a light microscope (EVOS XL Core, Thermo Fisher, Switzerland). NBT positive and negative cells were quantified using the trainable WEKA segmentation plugin within ImageJ analysis software [31].

### Flow cytometry

Cells were assessed for differentiation by flow cytometric analysis of myeloid maturation surface marker CD11b (#R0841, clone 2LPM19c, Dako, Glostrup, Denmark). Cells were analyzed using a BD SORP LSR II equipped with the BD FACSDiva software. For assessment of the viability, cells were stained with either AnnexinV-PE indicating activated apoptosis as well as DAPI for identification of dead cells or DAPI only. Reactive oxygen species were assessed by staining the cells with 2.5 µM CM-H2DCFDA (#C6827, Thermo Fisher Scientific) for total ROS or 2 µM of Mitosox dye (#M36008, Thermo Fisher Scientific) for measurement of mitochondrial superoxide. For ROS dyes, cells were stained for 25 min at 37 °C. Data points were analyzed using FlowJo software.

### InCell analysis of yH2AX foci

To quantify nuclear yH2AX foci, 1.5 × 10^5^ cells were grown over the treatment period in 96-wells of Ibidi optical 96-well plate (#89626, Ibidi GmbH, Planegg, Germany). Following treatment, cells were washed in PBS and fixed for 10 min at RT using 2% paraformaldehyde. Cells were permeabilized for 10 min using 0.5% Triton-X in PBS and blocked for 1 h in 10% BSA/PBS. Cells were incubated in primary antibody (#2577, Cell Signaling Technologies) at a dilution of 1:500 overnight at 4 °C. Cells were washed three times and stained with a FITC-conjugated secondary antibody (anti-rabbit, 111-096-045, Jackson ImmunoResearch). A nuclear mask was obtained via DAPI staining. Images were acquired using an InCell 2000 Analyzer automated microscope (GE Healthcare Life Sciences) from the Microscopy Imaging Center (MIC) at the University of Bern. Image analysis and quantification were performed using CellProfiler (Broad Institute) [32].

### Next-generation RNA sequencing (RNAseq) and assay for transposase-accessible chromatin using sequencing (ATACseq)

HL60 *HK3*-null and Cas9 expressing control cells were treated with either 1 µM of ATRA or DMSO control (0.01%). To keep cell death at a minimum, the treatment period was set to 36 h. In total, 2 × 10^5^ or 1 × 10^5^ cells from two to three biological replicates of each condition were flash-frozen and sent to Active Motif for subsequent RNAseq and ATACseq assays, respectively. Samples were sequenced in a paired-end, directional manner on an Illumina NextSeq 500 platform. Please see supplementary methods for data analysis.

### Mass spectrometric analysis of knockout panels and HL60 HiBiT pulldown

Knockout of HK2 and HK3 respectively was confirmed via MS analysis of pooled samples for two monoclonal populations per HK. Samples were treated with 1 µM ATRA (NB4) or 100 nM Vitamin D3 (HL60) for 4 days to induce HK3 expression.

Affinity purification beads were loaded on a 10 kDa cut-off filter (Vivacon 500, Sartorius) and centrifuged for 10 min at 6000 × *g* at 4 °C. Proteins were reduced with 1 mM dithiothreitol (DTT, Sigma) and alkylated using 5.5 mM iodoacetamide (IAA, Sigma) sequentially for 20 min incubation for each step in 8 M urea in 50 mM ammonium bicarbonate buffer, pH 7.5 (ABC buffer). Samples were centrifuged for 20 min at 6000 × *g*. To wash out detergent and urea, 200 μL ABC buffer were added to the filter and samples were centrifuged for 20 min at 6000 × *g* and repeated again for 30 min at 6000 × *g*. About 100 μL sequencing grade modified trypsin (Promega) in ABC buffer were added to samples and incubated at 37 °C overnight. Peptides were recovered by centrifugation at 21,100 × *g* and 200 μL ABC buffer were added to the filter and centrifuged for 10 min at 21,100 × *g*. 1% trifluoracetic acid (TFA, Sigma) was added to acidify the solution and inactivate trypsin. Peptides were purified on STAGE tips, which were equilibrated with buffer B and washed with buffer A (0.1% formic acid in water) beforehand, as described [33] and eluted using 50 μL buffer B (80% acetonitrile, 0.1% formic acid in water). Samples were concentrated with a SpeedVac vacuum concentrator (Thermo Fisher Scientific), and the final volume was adjusted to 20 μL using buffer A*/A (30% buffer A* with 3% acetonitrile and 0.3% TFA in water, and 70% buffer A). Samples were analysed on a Q Exactive Plus mass spectrometer, coupled to an EasyLC 1000 (Thermo Fisher Scientific), as described [34].

### Proteomic data analysis

Data were normalized to the median of each experiment and log2 was transformed. The iBAQ values were calculated as the sum of all peptide intensities divided by the number of observable peptides of a protein. Proteins with mean positive iBAQ values for at least one of the conditions were kept in the analysis. To find positively or negatively enriched proteins, the iBAQ values were analyzed by two-way ANOVA. After statistical analysis, proteins with a significant difference in their enrichment levels at *p* value < 0.05 and fold differences ≥1.3 were selected. Principal component analysis (PCA) analysis was used to map the variations among the analyzed samples. Data were clustered using the standard Euclidean method based on the average linkage and heatmaps were generated according to the standard normal distribution of the values.

### Gene ontology analysis

For gene ontology (GO) enrichment, the list of differentially expressed genes/proteins was grouped into functional hierarchies. Enrichment scores were calculated using a chi-square test comparing the proportion of the gene list in a group to the proportion of the background genes/proteins using the GO resource database (geneontology.org), KEGG mapping (www.genome.jp/kegg), and Enrichr database (amp.pharm.mssm.edu/Enrichr). A value of 3 or higher corresponded to a significant GO enrichment (*p* < 0.05).

### Gene set enrichment analysis

Gene set enrichment analysis (GSEA) was performed using GSEA software v.4 (Broad Institute, Cambridge). Enrichment analysis was assessed for all pathway-related genes obtained from the Pathcards database (pathcards.genecards.org) or from the Broad Institute’s Molecular Signatures Database (MSigDB).

### Statistical analysis

Nonparametric Mann–Whitney *U*-tests were applied to compare the difference between the two groups using Prism software. *P* values <0.05 were considered statistically significant.

## Results

### HK3 expression is predominantly linked to cells of myeloid origin

**Fig. 1:**
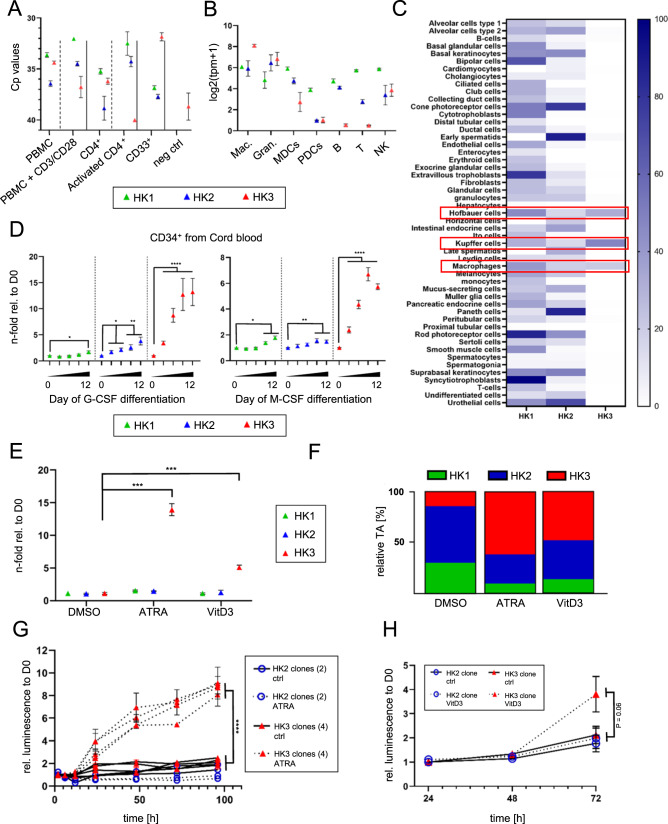
HK3 expression was predominant in myeloid tissue and markedly induced upon myeloid maturation. **A** mRNA expression (Cp values) of HK1–3 in PBMCs and specific isolated/activated subpopulations. 293 T cells were used as negative ctrl. (*n* = 2, two technical replicates each, three individual donors, mean ± SEM). **B** *HK1–3* transcript expression pattern across the hematopoietic cell lineages (Mac. macrophage lineage, Gran. granulocytic lineage, MDCs myeloid dendritic cells, PDCs plasmacytoid dendritic cells, B B cell lineage, T T cell lineage, NK NK cell lineage). Data accessed on the Haemoshpere Database. **C** The Human Protein Atlas *HK1–3* single-cell gene expression data in various cell types [35]. **D** *N*-fold relative expression of *HK1–3* mRNA levels during 12 days of in vitro neutrophil (G-CSF) or monocyte/macrophage (M-CSF) differentiation of CD34^+^ HSPCs isolated from human cord blood (*n* = 2, two technical replicates each, four individual donors, mean ± SEM). **E** *N*-fold relative *HK1–3* mRNA expression levels in HL60 cells after 6 days of either ATRA-induced neutrophil-like differentiation (*n* = 3) or Vitamin D_3_ (VitD_3_)-induced monocytic-like differentiation (*n* = 2). Data were expressed as mean ± SEM. Each experiment was performed with at least two technical replicates. **F** Relative transcript abundance as a percentage of total hexokinase expression in HL60 cells treated with either ATRA, VitD3, or DMSO control. Calculated as described here [56]. **G**, **H** Relative protein levels of HK2 and HK3 during 4 days of ATRA treatment (**G**) or 3 days of VitD_3_ (**H**) as measured by luminescent quantification of HiBiT-tag expression (**G**: *n* = 3, 4 individual clones for HK3-HiBiT, 2 individual clones for HK2-HiBiT. Mean ± SEM, Statistical analysis: two-way ANOVA. H: *n* = 2, 1 clone each). Mann–Whitney *U*: *p < 0.05, **p < 0.01, ***p < 0.001, ****p < 0.0001.

We determined *HK1–3* mRNA expression in whole peripheral mononuclear cells (PBMCs) populations, PBMCs depleted of CD33-expressing cells, CD33^+^ cells (representing the myeloid compartment), as well as purified CD4^+^ T cells. The isolated CD33^+^ lineage demonstrated the highest *HK3* mRNA levels (Fig. 1A). PBMCs that were activated with anti-CD3/CD28 beads in order to stimulate T cell expansion lost *HK3* expression. No *HK3* mRNA was detected in the purified, activated CD4^+^ T cell population while the non-activated control showed a signal close to our negative control.

Consistent with our findings, *HK3* expression in a publicly available dataset showed a roughly 100-fold higher expression in macrophages and granulocytes compared to cells of B or T cell origin (Figs. 1B and S1A). Low *HK3* mRNA levels were found in myeloid dendritic (CD14^−^, BDCA-2^−^, CD123^low/medium^) and NK lineages (CD56^+^, CD3^−^, CD19^−^). *HK1* and *HK2* had similar expression levels, with the exception of plasmacytoid dendritic cells, which showed low expression of *HK2*. Confirming our earlier report, there was a correlation between the expression of *HK3* and the myeloid transcription factor *PU.1* (*SPI1*), which was shown to regulate the expression of *HK3* [14] (Fig. S1B, Pearson correlation 0.83). A comparison of expression of HKs across tissues highlighted the hematopoietic predominance of HK3 (Fig. 1C, The Human Protein Atlas [35]). When taken together, our current and published findings [14, 35] confirm that *HK3* mRNA expression is clearly associated with myeloid lineage.

### HK3 is significantly induced during myeloid differentiation of both healthy and leukemic myeloid progenitor cells

Ex vivo differentiation of CD34^+^ progenitor cells using G- or M-CSF resulted in significant 10- (*p* < 0.0001) and 6-fold (*p* < 0.0001) induction of *HK3* mRNA levels during neutrophil or macrophage differentiation, respectively (Figs. 1D and S1C). In contrast to *HK3*, *HK1* mRNA expression increased slightly with 1.7- and 1.8-fold changes during neutrophil and macrophage differentiation. *HK2* mRNA increased 3.8-fold during neutrophil differentiation but remained relatively constant during macrophage differentiation with a 1.4-fold difference in expression from day 0 to day 12.

Inducing neutrophil-like differentiation with ATRA in HL60 cells resulted in 13.9-fold (*p* < 0.001) induction of *HK3* mRNA expression (Fig. 1E, left panels) paralleled by increased HK3 protein levels (Fig. S1D), whereas monocytic differentiation using VitD3 increased *HK3* mRNA levels fivefold (Fig. 1E, right panels). At a steady state in the absence of ATRA treatment, *HK3* only accounted for roughly 14% of total hexokinase expression assessed by calculation of relative transcript abundance, but after 6 days of ATRA treatment, *HK3* was responsible for 61% of total *HK* transcript abundance. The relative contribution of *HK1* and *HK2* as components of hexokinase abundance declined from 30 to 10% and 56 to 29%, respectively. A similar phenomenon was seen upon VitD_3_-induced monocytic differentiation (Fig. 1F).

We first tested the specificity of various commercial anti-HK3 antibodies using HEK 293 T cells transiently expressing HK1, HK2, or HK3 and found considerable cross-reactivity of HK3 antibodies to the other HKs [30]. In order to specifically confirm the upregulation of HK3 protein during myeloid differentiation, we tagged the *HK2* and *HK3* genome in coding sequences with an 11 amino acid HiBiT-tag in HL60 cells using CRISPR technology (Fig. S1E) [29, 30]. We assessed subcloned cell lines expressing HiBiT-tagged HK2 or HK3 proteins and found an 8–10-fold relative protein induction (*p* < 0.0001) of HK3 after 4 days of ATRA treatment (Figs. 1G and S1F) and a marked fourfold increase (*p* = 0.06) upon VitD_3_ differentiation (Fig. 1H). HK2 expression remained stable during ATRA or VitD_3_ treatment (Fig. 1G, H). Of note, lentiviral vector-mediated delivery of *HK2* or *HK3* transgenes to healthy donor CD34^+^ HSPCs to investigate the cellular effects of ectopic HK expression on myeloid differentiation failed due to cellular death of HK-ectopically expressing cells (Fig. S1G). These findings indicate that normal myeloid cells are sensitive to increased HK2 and HK3 protein levels.

### Loss of HK3 has no impact on G6P production or glycolytic activity in AML cells

**Fig. 2:**
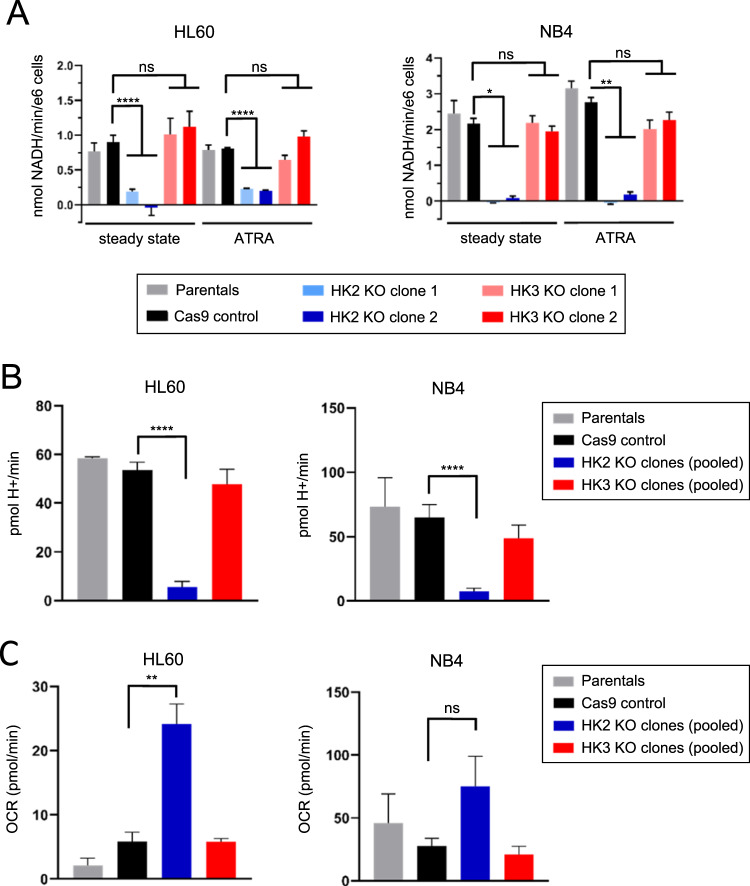
HK3 is dispensable for glucose-6-phosphate generation and glycolysis. **A** Hexokinase activity assay measuring G-6-P generation in HL60 and NB4 cell line panels at steady state and after 3 days of ATRA treatment (*n* = 3, 2 technical replicates each, mean ± SEM). **B**, **C** Seahorse analysis of *HK2* and *HK3* KO in HL60 and NB4 cells. Glycolytic rate assay (**B**) and mitochondrial stress test (**C**) measuring extracellular acidification rate as a function of glycolytic activity and oxygen consumption as a function of mitochondrial respiration, respectively (*n* = 3, 4 technical replicates each, mean ± SEM). Mann–Whitney *U*: *p < 0.05, **p < 0.01, ****p < 0.0001.

Clonal *HK2/3* KO cell lines were generated and confirmed via sequencing of genomic DNA and protein determination by mass spectrometry (Supplementary Table S2). Interestingly, disruption of *HK2* or *HK3* gene expression did not alter the expression of remaining hexokinases (Fig. S2A). Surprisingly, hexokinase activity assays performed on *HK2*- and *HK3*-null HL60 and NB4 cell lines showed that only loss of HK2 negatively affected G6P production (Fig. 2A, left panels), while loss of HK3 expression had no effects on glucose phosphorylation. Given that HK3 is weakly expressed at a steady state, we also performed HK activity assays after 3 days of ATRA treatment, when levels of HK3 normally have increased roughly sixfold. Regardless, phosphorylation of glucose was defective only in *HK2*- but not *HK3*-null cells (Fig. 2A, right panels).

While the loss of HK2 substantially abrogated extracellular acidification rate (ECAR) as a function of glycolytic activity in both HL60 and NB4 cell lines, loss of HK3 did not affect steady-state glycolytic activity (Fig. 2B). At the same time, basal respiration was comparable between control and *HK3*-null cells in both cell lines. Oxygen consumption rate (OCR) was increased several-fold in HL60 *HK2*-null cells, whereas it did not reach significance in NB4 cells (Fig. 2C). To evaluate whether the glycolytic activity was dependent on the high levels of glucose present in RPMI media (11 mM), the glycolytic rate assay was also performed after cells were maintained in MEMα medium (5.5 mM) for 3 days prior to the experiment to better reflect physiological glucose levels in human plasma [36]. Similar glycolytic effects were observed with 50% reduced glucose levels (Fig. S2B).

These results indicate that HK3 was dispensable for glycolytic activity in different AML cell lines, while HK2 was the glycolytically active HK isoform.

### HK3 supports AML cell survival during ATRA-induced myeloid differentiation

**Fig. 3:**
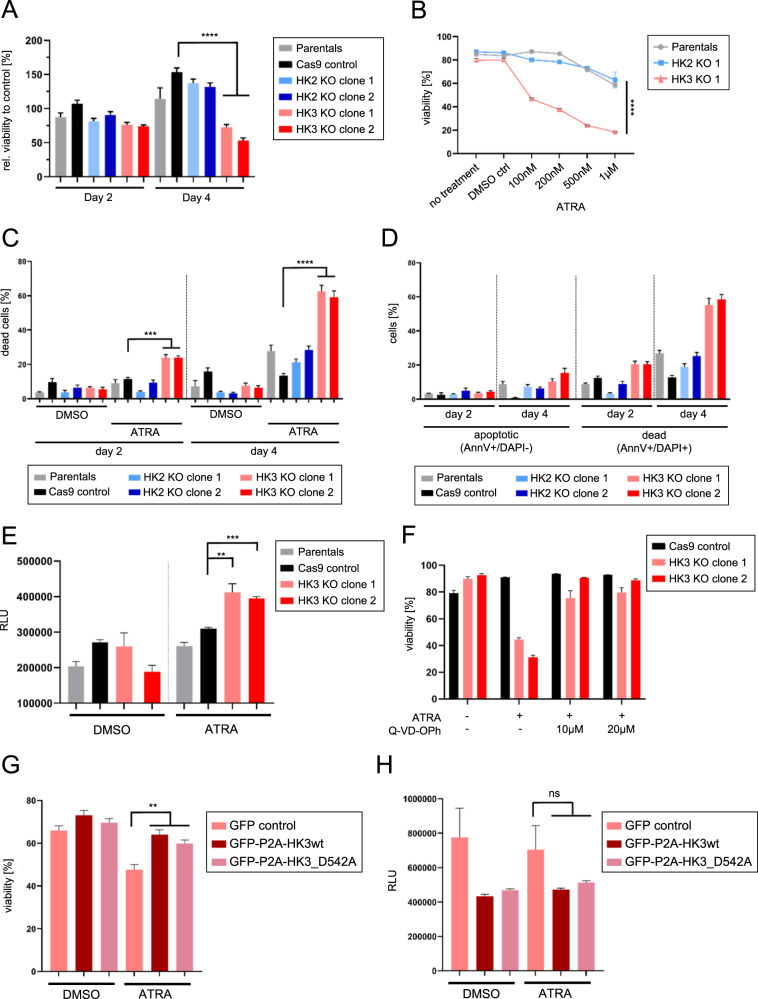
Loss of HK3 significantly increases ATRA-induced cell death. **A** Relative cell viability as measured by alamarBlue^®^ reduction capacity on day 2 and 4 of ATRA treatment. Normalized to untreated control (*n* = 2, 4 technical replicates each, mean ± SEM). **B** ATRA titration showing dose-dependent induction of cell death in *HK3*-null HL60 clone after 4 days of treatment (*n* = 2, 2 technical replicates each, mean ± SEM). **C** Flow cytometric analysis of viability in HL60 cell lines (DAPI exclusion) during 4 days of ATRA treatment (*n* = 5, 2 technical replicates each, mean ± SEM). **D** Flow cytometric analysis of apoptosis induction via AnnexinV staining during ATRA treatment of HL60 cell lines (*n* = 3, mean ± SEM). **E** Caspase −3/−7 activity in HL60 cell lines after 48 h of ATRA treatment (*n* = 2, 3–4 technical replicates each, mean ± SEM). **F** Flow cytometric analysis of HL60 cell viability (DAPI exclusion) upon 4 days of treatment of indicated cell lines with either DMSO control, ATRA alone, or ATRA in combination with pan-caspase inhibitor Q-VD-OPh at 10 or 20 µM (*n* = 2, 2 technical replicates each, mean ± SEM). **G**, **H** Flow cytometric analysis of viability on day 4 of ATRA treatment (*n* = 3, mean ± SEM) (**G**) as well as Caspase-3/−7 activity after 2 days of ATRA treatment (*n* = 2, 3 technical replicates each, mean ± SEM) (**H**) in NB4 *HK3*-null clone stably transduced with either GFP control, a GFP-P2A-HK3wt construct or a GFP-P2A-HK3 D542A mutant. GFP-expressing cells were FACS sorted prior to the experiment. Mann–Whitney *U*: **p < 0.01, ***p < 0.001, ****p < 0.0001.

Given published reports on a possible cytoprotective role for HK3 [14, 21] in AML cell lines, we investigated if complete loss of HK3 function in myeloid cells affects cellular viability rather than glycolysis. We found that the two *HK3*-null clonal cell lines upon ATRA treatment by day 4 showed 53 and 66% reduced viability as compared to controls (*p* < 0.0001, Figs. 3A). A titration of ATRA revealed a dose-dependent induction of cell death within *HK3*-null cells, with a 50% cell death seen after 4 days of treatment with a low concentration of 100 nM ATRA (Fig. 3B). We found that *HK3*-null cells showed high levels of cell death (60%, *p* < 0.0001) after 4 days of ATRA-mediated differentiation, while *HK2*-null cells displayed a lower level of cell death (20%) (Fig. 3C, D). Further investigation of apoptotic activity in *HK3*-null HL60 clones upon ATRA treatment demonstrated both enhanced Caspase-3/-7 activity (*p* = 0.0073 for clone 1, *p* = 0.0002 for clone 2, Fig. 3E) and cleavage of Caspase-3 (Fig. S3A). Furthermore, inhibition of caspase activity using the pan-caspase inhibitor Q-VD-OPh rescued ATRA-induced cell death in *HK3*-null clones (Fig. 3F). The cytotoxic effect resulting from the loss of HK3 upon ATRA treatment was confirmed in NB4 cells, although the phenotype was not as severe as found for HL60 *HK3*-null cells (Fig. S3B, C).

Given that loss of HK3 affected cellular viability in HL60 and NB4 upon ATRA treatment, we tested whether either wild-type HK3 or the partial enzymatically active HK3 D542A mutant [21] could rescue cellular viability. The D542A mutation resulted in a 44% reduction of hexokinase activity as compared to wild type when ectopically expressed in HEK 293 T cells (Fig. S3D). Lentiviral vectors were used to deliver wild type or the D54A mutant HK3 transgenes to NB4 and HL60 *HK3*-null cells. In NB4, both HK3 wild type and D54A mutant rescued cellular viability when treated with ATRA (Fig. 3G) and showed a similar trend of decreased Caspase activation (Fig. 3H). However, HK3 ectopic expression was not tolerated in HL60 HK3 null cells, thus not allowing rescue assessment. Taken together, our findings demonstrate a non-glycolytic function of HK3 in promoting AML cell viability during neutrophil differentiation.

### Loss of HK3 leads to an increase in ROS production and an accumulation of DNA damage in AML cells

**Fig. 4:**
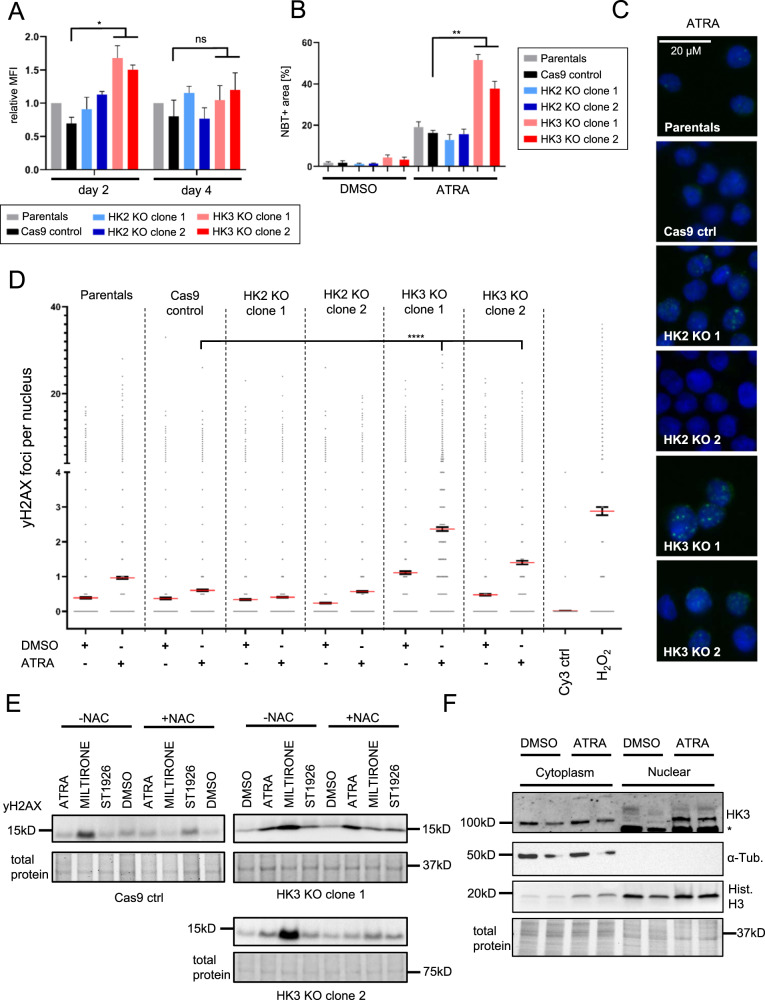
Loss of HK3 increases ROS and was correlated with DNA damage. **A** Relative MFI of CM-H2DCFDA staining in HL60 cell lines on day 2 and 4 of ATRA treatment (*n* = 2, 2 technical replicates each, mean ± SEM). **B** NBT positive cell area after 4 days of ATRA treatment in HL60 cell lines (*n* = 2, 2 technical replicates each, mean ± SEM). **C** Representative pictures of nuclear yH2AX foci in HL60 cell lines after 3 days of ATRA treatment (*n* = 2). **D** Quantification of nuclear yH2AX foci measured on an InCell microscope in HL60 cell lines ±3 days of ATRA treatment (*n* = 2, 500 nuclei/well, ≥2 wells per condition, mean ± SEM). **E** Western Blot analysis of yH2AX levels in HL60 control and *HK3*-null cell clones upon 24 h of indicated treatments. NAC samples were preincubated in 3 mM NAC for 2 h prior to adding treatments. **F** Nuclear fractionation of HL60 cell line ±4 days of ATRA treatment. *unspecific band at ~80 kDa. Mann–Whitney *U*: *p < 0.05, **p < 0.01, ****p < 0.0001.

*HK3*-null cells showed slightly increased total ROS levels as measured by flow cytometric analysis of the ROS cellular reporter CM-H2DCF-DA (*p* < 0.05 for HL60, ns for NB4) (Figs. 4A and S4A). Next, we identified a significant (*p* < 0.01) increase in superoxide anion levels (O_2_^-^) in *HK3*-null cells as compared to wild type and *HK2*-null cells using a nitroblue tetrazolium (NBT) assay (Fig. 4B). Mitochondrial ROS levels were not elevated in *HK3*-null lines (Fig. S4B).

We next asked whether this increase in ROS was associated with DNA damage. Given that ATRA treatment is not known to induce substantial amounts of DNA damage, we chose to use the detection of phosphorylated histone 2AX (yH2AX) as a sensitive marker of DNA double-strand break (DSB). In both HL60 and NB4 *HK3*-null cells, yH2AX signals increased when cells were depleted of HK3 but not with the loss of HK2 (Fig. S4C). To determine the presence of phosphorylated H2AX within the nucleus, we performed automated microscopy on *HK3*-null cells to quantify yH2AX positive foci within nuclei (Fig. 4C). While there were minor increases in yH2AX foci in parental, CAS9, and HK2-null cells upon ATRA treatment, the accumulation of foci was slightly elevated in *HK3*-null cells at a steady state, with significant increases after ATRA treatment (Fig. 4D). Of note, yH2AX foci were not resolved after a 24 h of rest period indicating impaired DNA repair in *HK3*-null cells (Fig. S4D). We next evaluated exposure of HL60 cell lines to other ROS-inducing agents such as Miltirone [37] or the atypical retinoid ST1926 [38]. Both compounds resulted in increased DNA damage in *HK3*-null cells as compared to controls (Figs. 4E and S4E). Interestingly, the DNA damaging effect of Miltirone was partially rescued by preincubation with ROS inhibitor *N*-acetylcysteine (NAC) but NAC did not rescue damage caused by either ATRA or ST1926 (Fig. 4E). Preincubation with NAC did not result in an increase in viability in *HK3*-null cells, as well as negatively affected the viability of Cas9 control cells (Fig. S4F). Given the panoply of cellular effects seen in *HK3*-null cells, we next asked whether HK3 might localize to the nucleus contributing to cellular regulation. Indeed, we detected a signal for HK3 within the nuclear fraction of both ATRA treated and untreated HL60 cells (Fig. 4F). Confocal imaging of HEK 293 T cells ectopically expressing FLAG-tagged HK2 or HK3 further identified a potential nuclear localization of both HK2/3 (Fig. S4G).

In total, these findings are supportive of a role for HK3 in promoting DNA protection, which appears to be independent of its reported function in glycolysis.

### Transcriptomic analysis identifies activation of apoptosis, enhanced reactive oxygen species as well as DNA damage signatures in AML *HK3*-null cells

**Fig. 5:**
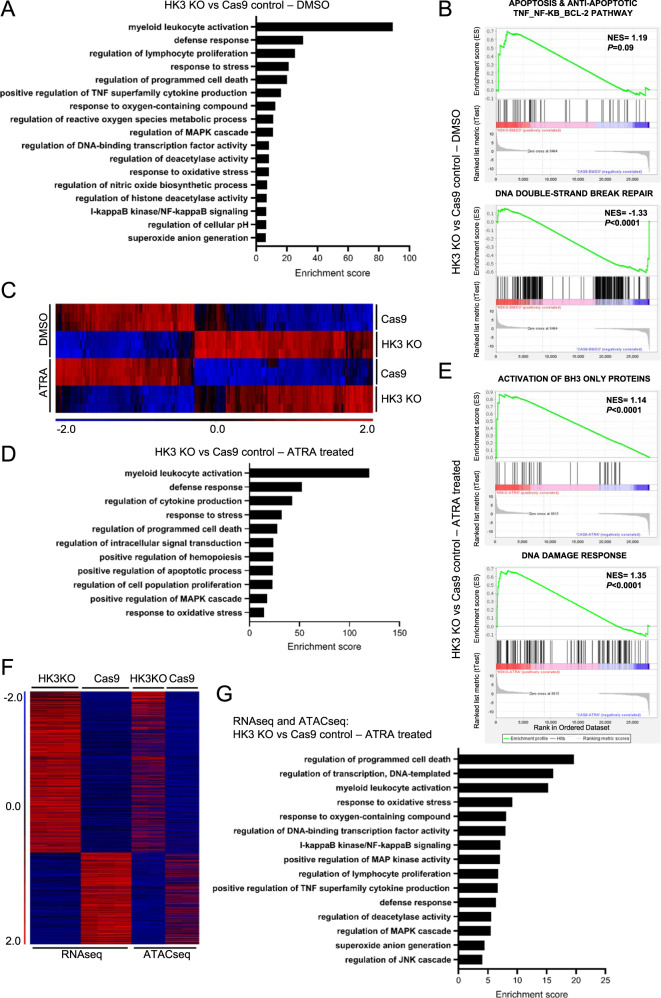
RNAseq and ATACseq analysis reveal enrichment and increased accessibility of cell death and stress pathways in *HK3*-null AML cells. **A** Gene ontology (GO) analysis of upregulated genes (*HK3*-null vs Cas9 control). **B** Gene set enrichment analysis (GSEA) represents the normalized enrichment score (NES) of indicated gene sets. **C** Heatmap of differentially expressed genes in DMSO and ATRA-treated *HK3*-null and control samples. **D** Gene ontology (GO) analysis of upregulated genes in ATRA-treated samples (*HK3*-null vs Cas9 control). **E** Gene set enrichment analysis (GSEA) represents the normalized enrichment score (NES) of indicated gene sets in *HK3*-null vs Cas9 control after ATRA treatment. **F** Heatmap of differentially regulated genes across RNAseq and ATACseq datasets among ATRA-treated samples of *HK3*-null and Cas9 control cells. **G** Gene ontology (GO) analysis of genes upregulated in both RNAseq and ATACseq analysis under ATRA treatment (*HK3*-null vs Cas9 control).

To gain insights into how the loss of HK3 might genetically connect to DNA damage and cell death pathways, we assessed the global and specific changes in the cellular transcriptome upon loss of HK3. RNA sequencing was performed with HL60 *HK3*-null and Cas9 control cell clones treated with either 1 µM ATRA or DMSO, as a diluent control. Principal component (PCA) and fold change analyses showed significant global transcript differences between untreated and ARTA treated HL60 *HK3*-null and Cas9 cells (Fig. S5A, B). GO analysis of gene sets showed differentially regulated genes between *HK3*-null and Cas9 control cells which at cellular steady state showed enrichment in myeloid/leukocyte activation gene sets and various stress responses gene sets, as well as regulation of programmed cell death and response to oxidative stress (Figs. 5A and S5C). The unbiased GSEA analyses provided an insight of gene sets from the *HK3*-null cells with significant enrichment of “detoxification of reactive oxygen species”, “apoptosis and anti-apoptotic TNFS_NF-kB_BCL-2 pathway”, as well as activation of “BH3-only proteins”. In contrast, “DNA double-strand break repair” was negatively enriched (Figs. 5B and S5D). Comparing hierarchical clustering for control and ATRA-treated samples revealed that global changes in gene expression between *HK3*-null cells and Cas9 control are independent of ATRA treatment (Figs. 5C and S5E). We next performed GO analysis of differentially regulated genes within the ATRA-treated cell samples and again determined that *HK3*-null cells are highly enriched for genes involved in defense responses, programmed cell death, and response to oxidative stress (Fig. 5D). Additional GSEA analysis of the datasets determined that enrichment of activation of BH3-only proteins, as well as DNA damage responses, were found in samples from *HK3*-null cells (Fig. 5E). Insights as to the loss of HK3 regulation on specific cellular pathways were highlighted by the finding that stress response and apoptotic pathways were enriched in both DMSO and ATRA-treated *HK3*-null as compared to Cas9 control. Thus, the loss of HK3 appears to significantly alter the normal cellular pathway architecture that results in decompensation and cell death when ATRA was added.

### Loss of HK3 increases chromatin accessibility of stress response and apoptotic pathways

We next asked whether loss of HK3 would alter chromatin accessibility within stress-related gene pathways. ATAC sequencing (ATACseq) and analysis demonstrated unique signatures in *HK3*-null as compared to Cas9 controls without or with ATRA treatment (Fig. S5F, G). Hierarchical clustering of differentially regulated genes between *HK3*-null and Cas9 control cells within our RNAseq and ATACseq datasets correlated well between the two approaches (Fig. 5F). Significantly upregulated genes in both datasets, RNAseq and ATACseq, were queried for GO terms and, among others, showed prominent enrichment for regulation of programmed cell death and response to oxidative stress in *HK3*-null cell samples (Fig. 5G). These findings are consistent with an undescribed function of HK3 participating in cellular pathways which appear to control chromatin accessibility in myeloid cells and that may protect differentiating cells from cell death.

### Pulldown of HK3 cellular extracts identifies the proapoptotic BCL-2 family member BIM as a direct interaction partner

**Fig. 6:**
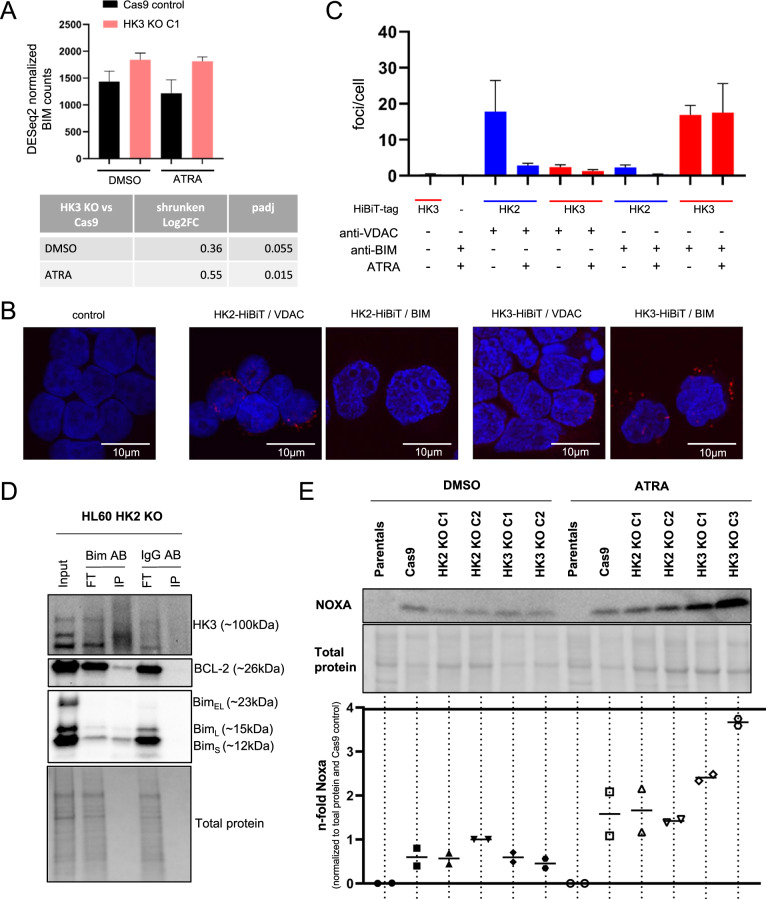
HK3 interactome reveals interaction with BH3-only protein BIM. **A** DESeq2 normalized counts and differential expression analysis of BIM transcript expression in *HK3*-null cells and control ±ATRA. Data represent the mean of three biological replicates. **B** Confocal imaging of proximity ligation assay (PLA) using either endogenously HiBiT-tagged HK2 or HK3 HL60 cell lines. Fluorescent foci indicate the colocalization of proteins. Primary antibodies used were mouse anti-HiBiT, combined with rabbit anti-VDAC or rabbit anti-BIM. Control: secondary antibodies only. **C** PLA quantification. Negative controls: First column, secondary antibodies only, second column HL60 not expressing a HiBiT-tag (*n* = 2, 2 technical replicates each, mean ± SEM). Anti-VDAC was used as a positive control for HK2 interactions. **D** Co-IP of HK3 and BIM. IP was performed in HL60 *HK2*-null cells treated with ATRA for 2 days using an anti-BIM antibody immobilized on Protein A/G magnetic beads. HK3, BCL-2, and BIM immunoblotting of flowthrough (FT) and pulled-down (IP) proteins are shown. BCL-2, a known BIM interacting protein, was used as a positive control. **E** NOXA western blotting of HL60 control, *HK2*- and *HK3*-null cell lines treated for 4 days with DMSO or ATRA. Quantification of NOXA protein expression of two independent experiments is shown below. NOXA expression was normalized to total protein and Cas9 control cell expression.

Given the increasing reports of glycolytic enzymes regulating cellular processes through functions outside the realm of metabolic activity, we asked whether HK3 is directly involved in regulating apoptotic or ROS-generating pathways. Mass spectrometry analysis of HiBiT-HK3 pulldown of extracts from HL60 cells identified 25 proteins participating in apoptosis signaling, RNA splicing, and various receptor signaling pathways (Fig. S6A). Among the top interacting hits, we identified the BH3-only protein BIM, also known as BCL2L11, a proapoptotic BCL-2 family member [39–41] Coincidently, our transcriptomic analysis had also previously revealed gene set enrichment for activation of BH3-only proteins (Fig. 5E). While levels of BIM were upregulated in both DMSO and ATRA-treated *HK3*-null versus Cas9 control cells in our RNAseq dataset (Fig. 6A), western blot analysis of BIM protein isoforms showed similar levels of BIM in *HK3*-null and wild-type cells in DMSO conditions (Fig. S6B). Using our HiBiT-tagged HK3 HL60 cell lines for a proximity ligation assay, we confirmed colocalization of HK3 and BIM whereas BIM did not colocalize with HiBiT-HK2 (Fig. 6B, C). HK2 is known to bind to the voltage-dependent anion channel (VDAC) at the mitochondrial outer membrane (MOM) [17], and we were able to confirm HK2 and VDAC colocalization in our HiBiT-tagged HK2 HL60 samples, demonstrating the suitability of our methodology for interrogating HK interacting cellular proteins. Interestingly, HK2 association with VDAC, which is known to confer cytoprotection [42], was markedly decreased under ATRA treatment. Moreover, VDAC did not colocalize with HK3. In order to validate our findings showing HK3 and BIM interaction, we performed BIM IP in HL60 HK2 knockout cells treated for 2 days with ATRA. While we could not detect any HK3 in the IP of the IgG control, we co-immunoprecipitated HK3 together with BIM. BCL-2 further served as a positive control for BIM interaction (Fig. 6D).

Interestingly, our RNAseq findings revealed additional BH3-only gene expression patterns, PUMA, BMF, and NOXA in *HK3*-null cells as compared to Cas9 control cells (Fig. S6C). We focused on NOXA and PUMA and determined that the NOXA protein expression was markedly upregulated in HL60 HK3 but not HK2 KO cells upon ATRA treatment (Fig. 6E). In contrast, PUMA protein levels did not show a consistent expression pattern between the different *HK3*-null cell clones nor between HL60 parental and Cas9 control cells (Fig. S6D).

Our findings strongly support the notion that HK2 and HK3 are part of distinct cellular pathways that regulated cell death. The findings further support an HK3-mediated cytoprotective mechanism through direct interaction with BIM and possibly additional proapoptotic BH3-only proteins during ATRA-induced differentiation of the leukemic cell line HL60 (Fig. S6E).

## Discussion

While all HKs have substrate binding sites in both the C- and N-terminal halves, HK1 and HK3 demonstrate catalytic activity only in their C-terminal half [43]. In contrast, HK2 is catalytically active in both the C- and N-termini [44]. Furthermore, HK3 differs from both HK1 and HK2 regarding the K*i* for the G6P, ~10-fold higher, while also showing inhibition of its activity at high glucose concentrations. Based on our findings, we posit that the predominant myeloid expression pattern of HK3, along with its distinct molecular features, implicate a more specialized and lineage-specific cellular role, as compared to the ubiquitously expressed HK1 and 2 isoforms. We have previously reported on the upregulation of HK3 during terminal differentiation of selected acute myeloid leukemia cell lines, specifically during ATRA-induced neutrophil-like differentiation of acute promyelocytic cells [14]. We now provide further insights to our original findings of HK3 myeloid expression and demonstrate that HK3 is greatly upregulated during in vitro myeloid differentiation of healthy CD34^+^ hematopoietic stem and progenitor cells.

Abrogation of *HK3* gene expression did not impact G6P generation or total glycolytic activity, in contrast, disruption of HK2 gene expression abrogated G6P levels as well as glycolysis. Moreover, HK3 did not compensate for the loss of HK2 function. ATRA treatment of HL60 and NB4 cells increased the levels of HK3 substantially, but surprisingly *HK2*-null cells expressing normal levels of HK3 still showed a major decrease in G6P levels. Our findings are consistent with the proposal that HK3 is dispensable for glycolytic activity in our AML cell line models.

Mitochondrially-bound hexokinases (HK1 and HK2) were previously shown to be cytoprotective and their association with mitochondria increases viability and survival. The binding of HK1 and 2 to the voltage-dependent anion channel at the mitochondrial outer membrane restricts access to the proapoptotic BCL-2 family member BAX and prevents apoptosis induction [42, 45]. Apart from HK1 and HK2, HK4 has also been found to play a role in balancing apoptotic activity as it can reside in a complex with proapoptotic BCL-2 family member BAD, whose phosphorylation status is dependent on glucose availability [46].

Based on the totality of our findings, we propose a role for HK3 in the regulation of cell death in myeloid cells. The precise mechanism(s) remains unknown, yet our present studies offer possible insights. ROS levels play a central role in cellular homeostasis and DNA integrity, yet are important signaling factors that have been shown to induce differentiation of HSCs and accelerate ATRA-induced differentiation of NB4 cells [47, 48]. The loss of HK3 leads to an increase in ROS, most likely through the increase of superoxide anions, however, the source of elevated ROS generation within *HK3*-null cells remains to be determined. In line with recent reports suggesting that HK2 interacts with proteins of the DNA damage repair machinery [49], we also found increased DNA damage in *HK3*-null cells upon various treatments. Interestingly, the preincubation with ROS scavenger NAC did not protect DNA integrity during neutrophil differentiation, nor was the damage resolved after a 24 h rest period. This may suggest that DNA damage in *HK3*-null cells could occur in a ROS-independent fashion and that the DNA damage repair mechanism(s) is impaired.

RNAseq and ATACseq analyses of *HK3*-null cells both confirmed the activation of programmed cell death and stress response pathways. These pathways were differentially regulated in *HK3*-null cells at steady state and was independent of the exogenous induction of cell death. Therefore, loss of HK3 likely conferred cellular pathway alterations, which with ATRA treatment resulted in decompensation and pronounced induction of apoptosis. Interestingly, we identified the BH3-only protein BIM as a direct interaction partner of HK3. BIM has previously been linked to neutrophil cell death and shortens myeloid life span [50, 51]. Additionally, in a Spi-1/Pu.1 transgenic mouse model where mice develop erythroleukemia, it has also been shown that Bim levels are downregulated through Pu.1 activity, as Pu.1 binds the Bim promoter and promotes repressive H3K27 trimethylation [52]. Furthermore, BIM has been shown to be a key effector in acute oxidative damage-induced apoptosis in AML cells [53]. The precise nature of this novel HK3-BIM interaction warrants further investigation. Apart from regulation through expression, BIM has been shown to possess multiple regulatory phosphorylation sites [54]. While some promote enhanced apoptotic activity, others appear to increase proteasomal degradation [54, 55]. With HK3 possessing kinase activity, a potential BIM regulation via HK3-mediated protein phosphorylation may represent a possible regulatory interaction.

Whether HK3 is crucial for healthy myeloid differentiation and whether it interacts with BIM or additional proapoptotic BH3-only proteins such as NOXA during myeloid development warrants further investigations and presents a limitation of the present study. As dynamic regulation of cell death and survival of myeloid cells is critical for maintaining adequate immune responses, an HK3-mediated regulation of myeloid life span could prove clinically relevant. Our findings support a prominent non-canonical cytoprotective function of HK3 during cellular differentiation of myeloid leukemic cells.

## Supplementary information

Supplementary Tables

Supplementary Figure Legends

Supplementary Figures

Supplementary Information

Original western blots

Reproducibility checklist

Agreement co-authors

## Data Availability

The datasets used and analyzed in this study are either publicly available or are included in this published article and its supplementary information files.
