# Supplementary material for: Hexokinase 3 enhances myeloid cell survival via non-glycolytic functions
Source: Cell Death Dis. 2022 May 11;13(5):448. doi: 10.1038/s41419-022-04891-w (PMC9091226; doi:10.1038/s41419-022-04891-w)
Supplement: Supplementary file 1 — Supplementary Tables [file 41419_2022_4891_MOESM1_ESM.pptx]

## Slide 1
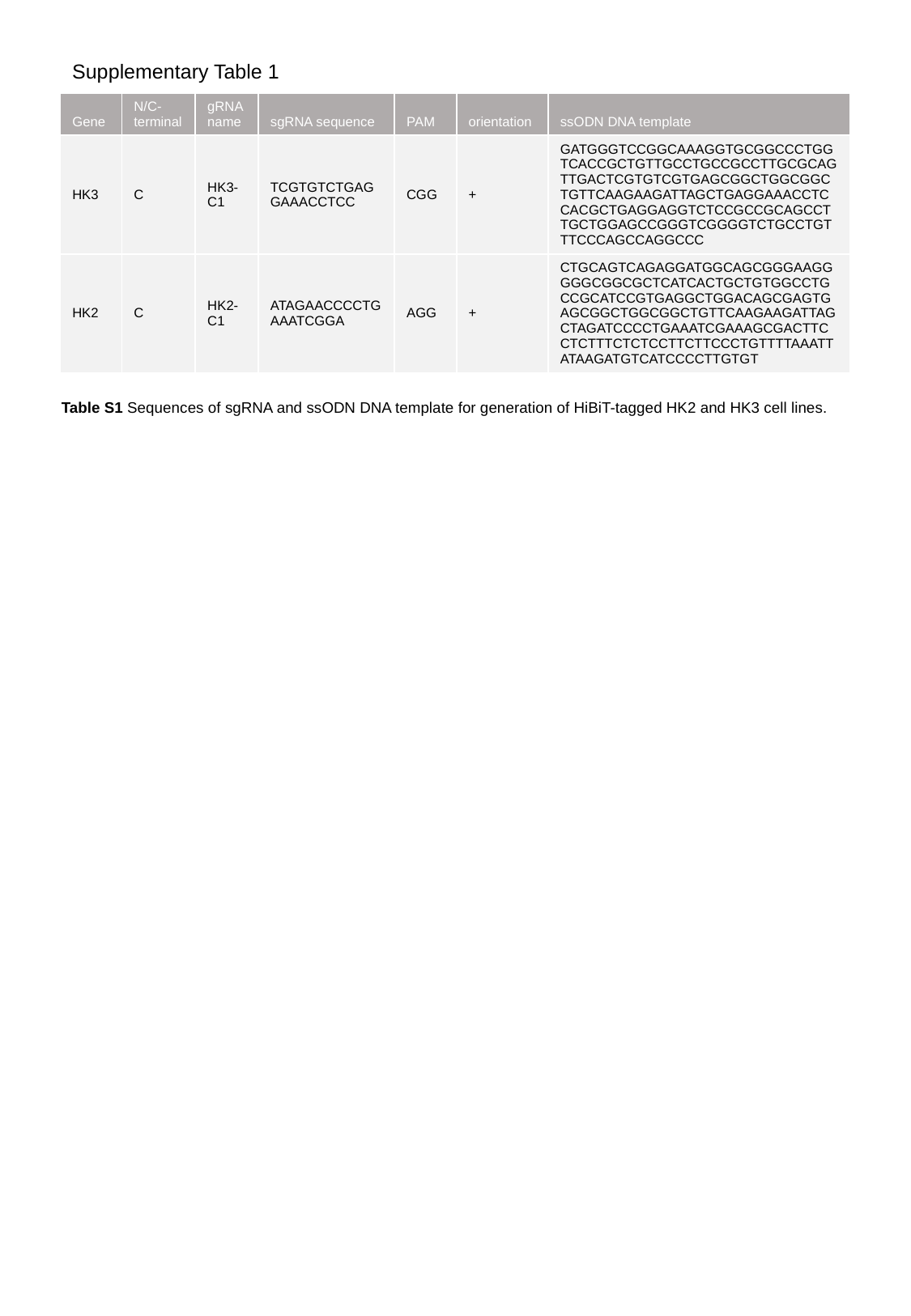

Supplementary Table 1
| Gene | N/C-terminal | gRNA name | sgRNA sequence | PAM | orientation | ssODN DNA template |
| --- | --- | --- | --- | --- | --- | --- |
| HK3 | C | HK3-C1 | TCGTGTCTGAGGAAACCTCC | CGG | + | GATGGGTCCGGCAAAGGTGCGGCCCTGGTCACCGCTGTTGCCTGCCGCCTTGCGCAGTTGACTCGTGTCGTGAGCGGCTGGCGGCTGTTCAAGAAGATTAGCTGAGGAAACCTCCACGCTGAGGAGGTCTCCGCCGCAGCCTTGCTGGAGCCGGGTCGGGGTCTGCCTGTTTCCCAGCCAGGCCC |
| HK2 | C | HK2-C1 | ATAGAACCCCTGAAATCGGA | AGG | + | CTGCAGTCAGAGGATGGCAGCGGGAAGGGGGCGGCGCTCATCACTGCTGTGGCCTGCCGCATCCGTGAGGCTGGACAGCGAGTGAGCGGCTGGCGGCTGTTCAAGAAGATTAGCTAGATCCCCTGAAATCGAAAGCGACTTCCTCTTTCTCTCCTTCTTCCCTGTTTTAAATTATAAGATGTCATCCCCTTGTGT |
Table S1 Sequences of sgRNA and ssODN DNA template for generation of HiBiT-tagged HK2 and HK3 cell lines.

## Slide 2
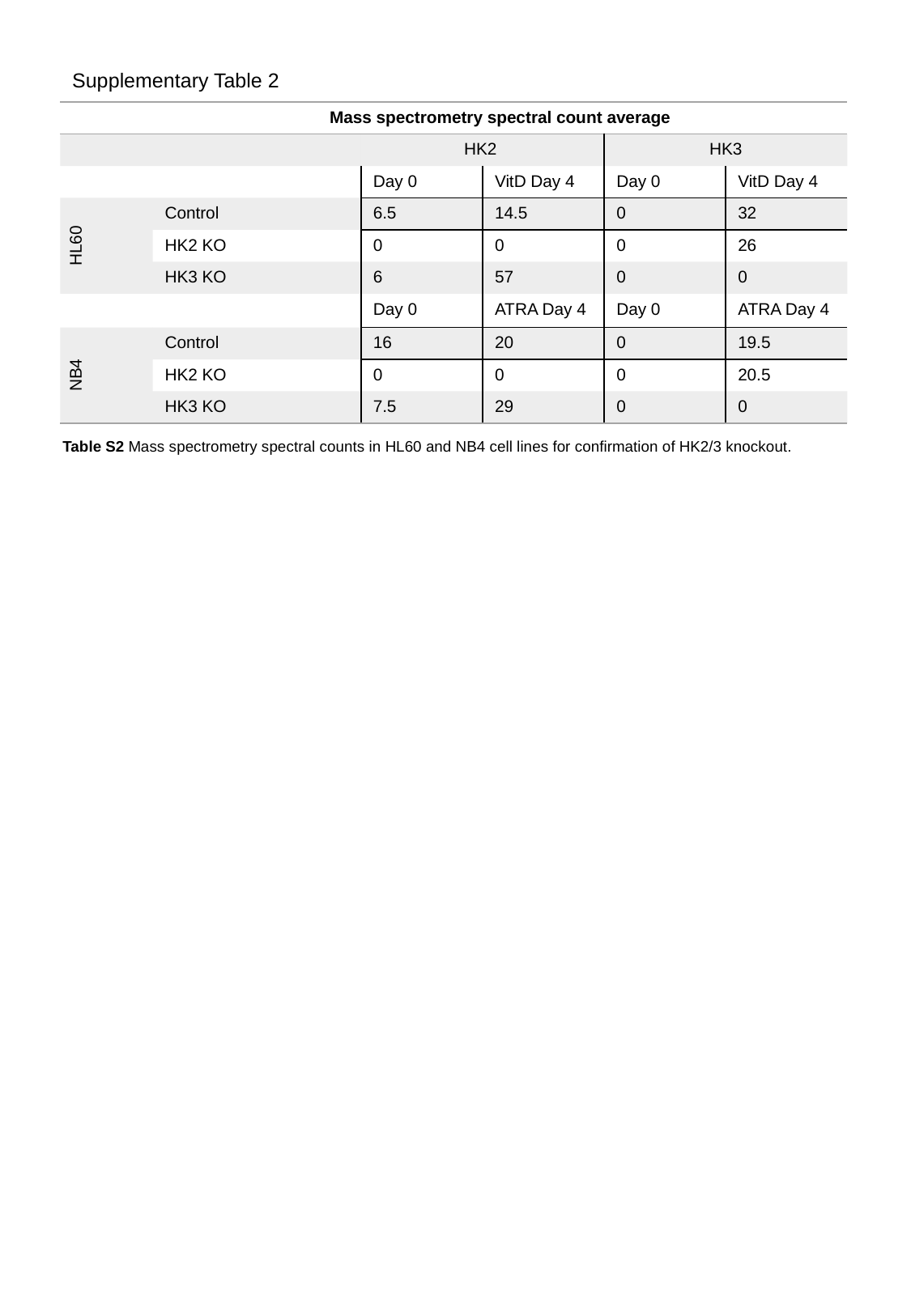

Supplementary Table 2
| | Mass spectrometry spectral count average | | | | |
| --- | --- | --- | --- | --- | --- |
| | | HK2 | | HK3 | |
| | | Day 0 | VitD Day 4 | Day 0 | VitD Day 4 |
| HL60 | Control | 6.5 | 14.5 | 0 | 32 |
| | HK2 KO | 0 | 0 | 0 | 26 |
| | HK3 KO | 6 | 57 | 0 | 0 |
| | | Day 0 | ATRA Day 4 | Day 0 | ATRA Day 4 |
| NB4 | Control | 16 | 20 | 0 | 19.5 |
| | HK2 KO | 0 | 0 | 0 | 20.5 |
| | HK3 KO | 7.5 | 29 | 0 | 0 |
Table S2 Mass spectrometry spectral counts in HL60 and NB4 cell lines for confirmation of HK2/3 knockout.
