# Supplementary material for: Hexokinase 3 enhances myeloid cell survival via non-glycolytic functions
Source: Cell Death Dis. 2022 May 11;13(5):448. doi: 10.1038/s41419-022-04891-w (PMC9091226; doi:10.1038/s41419-022-04891-w)
Supplement: Supplementary file 2 — Supplementary Figure Legends [file 41419_2022_4891_MOESM2_ESM.docx]

**Supplementary Information**

**Hexokinase 3 enhances myeloid cell survival via non-glycolytic functions**

Kristina Seiler^1,2,3^, Magali Humbert^1^, Petra Minder^3^, Iris Mashimo^3^, Anna M. Schläfli^1^, Deborah Krauer^1^, Elena Federzoni^3^, Bich Vu^4^, James J. Moresco^5^, John R. Yates III^5^, Martin C. Sadowski^1^, Ramin Radpour^6,7^, Thomas Kaufmann^8^, Jean-Emmanuel Sarry^9,10^, Joern Dengjel^4^, Mario P. Tschan^1,2,*^ and Bruce E. Torbett^3,11,12,13*^

**Supplementary Figure Legends**

**Figure S1.** **A** HK1-3 gene expression in specific hematopoietic populations. Data accessed via Bloodspot database. **B** Pearson correlation between PU.1 and HK3 expression within hematopoietic lineages. Data accessed via Haemosphere database. **C** N-fold relative expression of HK1-3 mRNA levels during 12 days of *in vitro* neutrophil (G-CSF) differentiation of CD34^+^ HSPCs isolated from human mobilized peripheral blood (One donor, biological replicates n=2, measured twice). HK1 (circles), HK2 (quares), and HK3 (triangles). **D** Western blot analysis of HK3 protein levels after 4 days of ATRA treatment in HL60 cells (n=2). **E** Illustration of HiBiT-tagging of endogenous proteins. **F** Levels of endogenous HK2-HiBiT or HK3-HiBiT in tagged HL60 cell lines ±3 days of ATRA treatment. Assessed via anti-HiBiT mAB. **G** GFP expression in CD34^+^ primary HSPCs transduced with GFP only control or GFP-P2A-HK constructs during G-CSF or M-CSF differentiation. While control construct was transduced at high levels and stably expressed, HK2 and HK3 construct transduction was not tolerated (n=2, 4 individual donors).

**Figure S2.** **A** Western blot analysis of HK1 (HL60) and HK2 (NB4) levels in respective HK3 and HK2 KO cells. **B** Seahorse analysis of basal glycolytic activity in HL60 and NB4 cell lines cultured in MEMα (n=3, p<0.0001, mean±SEM).

**Figure S3. A** Cleaved Caspase 3 western blotting of HL60 control, HK2 and HK3 KO cell lines treated for 4 days with DMSO or ATRA. Quantification of clCaspase 3 protein expression of two independent experiments is shown below. ClCaspase 3 expression was normalized to total protein and CAS9 control cell expression. **B** Flow cytometric analysis of cell death induction via DAPI staining during ATRA treatment of NB4 cell lines (n=3, p<0.001, mean±SEM). **C** Caspase -3/-7 activity in NB4 cell lines after 48h of ATRA treatment (n=2, p<0.001, mean±SEM). **D** Hexokinase activity in HEK 293T cell lines overexpressing either GFP only, GFP-P2A-HK3 wild type or D542A kinase domain mutant HK3 (n=2, 2 technical replicates each, p<0.05).

**Figure S4. A** Relative MFI of CM-H2DCFDA staining in NB4 cell lines on day 2 and 4 of ATRA treatment (n=2, 2 technical replicates each, mean±SEM). **B** Mitochondrial ROS content in HL60 cell lines after 2 days ±ATRA treatment measured via MFI of MitoSOX staining (n=2, 2 technical replicates each, mean±SEM). **C** Western Blot analysis of yH2AX levels in HL60 (upper) and NB4 (lower) cell lines ±2 days of ATRA treatment *=unspecific band. **D** Quantification of nuclear yH2AX foci measured on an InCell microscope in HL60 cell lines after 2 days of ATRA treatment followed by a 24h rest period in absence of ATRA (n=2, mean±SEM). **E** Western Blot analysis of yH2AX levels in HL60 cell lines ±24h of Miltirone or ST1926. **F** Relative cell viability assessment by alamarBlue® reduction capacity in control (Cas9), HK2 and HK3 KO cells upon ATRA treatment alone or in combination with NAC for 24h. **G** Confocal images of HEK 293T cells ectopically expressing FLAG-tagged HK2 or HK3. Cells were stained using anti-FLAG antibody (#F3165, Sigma) and DAPI. Images were captured on a Zeiss 780 confocal microscope and processed using Image J software.

**Figure S5. A** Principal component analysis of transcriptomic data of HK3-null samples and control ±ATRA (n=3). **B** Volcano plot representation of differentially expressed genes in HK3-null versus Cas9 control in DMSO control condition (fold change>2 and FDR<0.05).**C** Heatmap of differentially regulated genes in HK3-null vs control in DMSO control condition. **D** GSEA analysis representing the normalized enrichment score (NES) of indicated gene sets in HK3-null vs Cas9 control in DMSO control condition. **E** Volcano plot representation of differentially expressed genes in HK3-null versus Cas9 control after ATRA treatment (fold change>2 and FDR<0.05). **F** Number of differentially regulated chromatin regions in pairwise comparison of indicated samples showing largest difference in HK3-null vs control in DMSO control condition. **G** GO analysis of more accessible gene regions in HK3-null compared to Cas9 control upon ATRA treatment.

**Figure S6. A** Volcano plot of proteins identified in MS analysis of endogenous HiBiT-HK3 pulldown in ATRA treated samples (n=3). **B** Western blot analysis of BIM isoform levels in HL60 control and HK2 or HK3 null cell lines ±2 days of ATRA treatment. **C** Heatmap of differentially regulated BH3-only genes in HK3-null vs control in DMSO control or ATRA treatment conditions. **D** PUMA western blotting of HL60 control, HK2- and HK3-null cell lines treated for 4 days with DMSO or ATRA. Quantification of PUMA protein expression of two independent experiments is shown below. PUMA expression was normalized to total protein and Cas9 control cell expression. E Illustration of proposed mechanism of cell death in HK3-null AML cell lines upon ATRA treatment.
