# Supplementary material for: Hexokinase 3 enhances myeloid cell survival via non-glycolytic functions
Source: Cell Death Dis. 2022 May 11;13(5):448. doi: 10.1038/s41419-022-04891-w (PMC9091226; doi:10.1038/s41419-022-04891-w)
Supplement: Supplementary file 3 — Supplementary Figures [file 41419_2022_4891_MOESM3_ESM.pptx]

## Slide 1
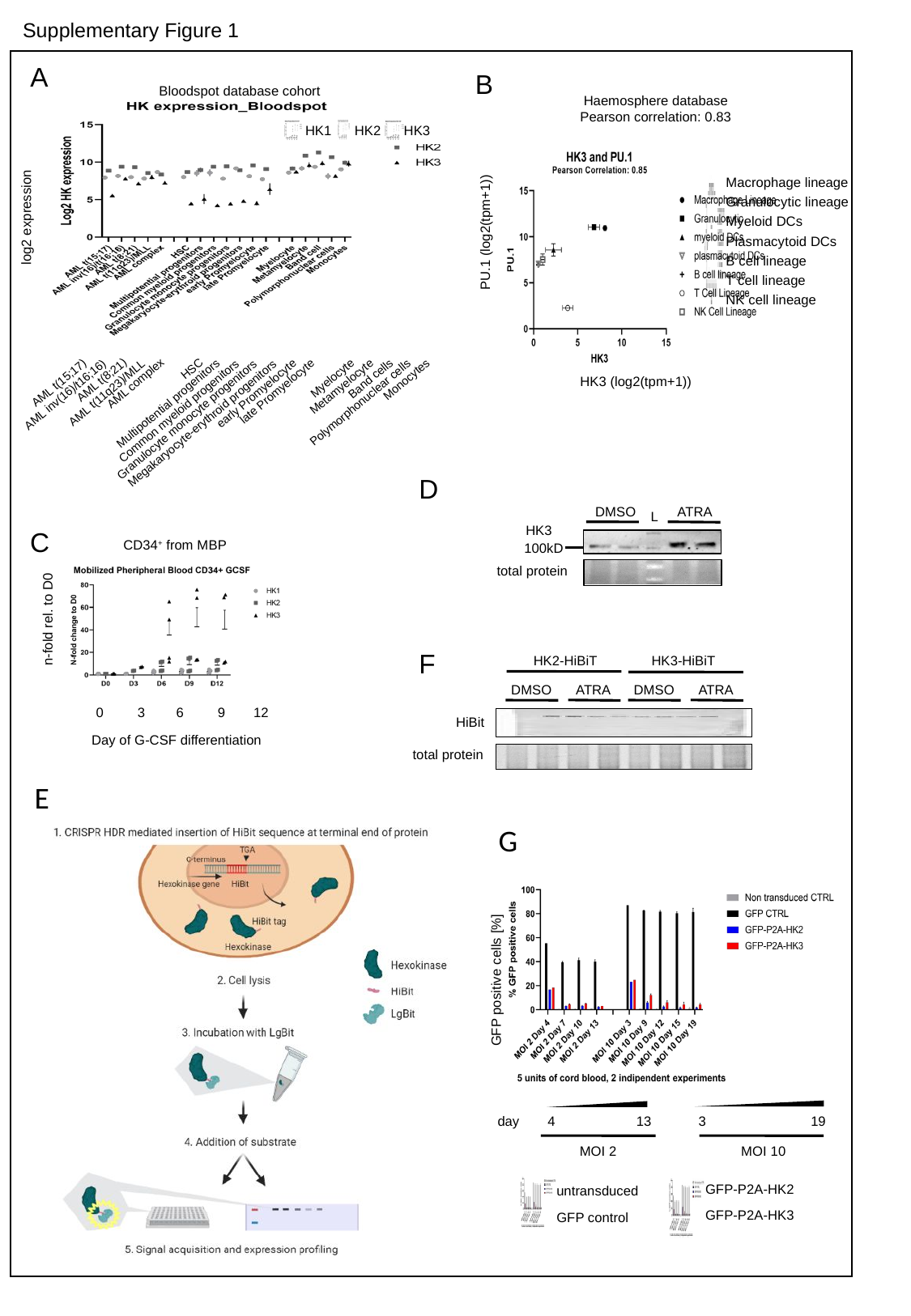

Supplementary Figure 1
A
B
Bloodspot database cohort
Haemosphere database
Pearson correlation: 0.83
HK1 HK2 HK3
Macrophage lineage
Granulocytic lineage
Myeloid DCs
Plasmacytoid DCs
B cell lineage
T cell lineage
NK cell lineage
log2 expression
PU.1 (log2(tpm+1))
HSC
Myelocyte
Band cells
Monocytes
HK3 (log2(tpm+1))
AML t(15;17)
Metamyelocyte
late Promyelocyte
AML t(11q23)/MLL
early Promyelocyte
AML inv(16)/t16;16)
AML complex
AML t(8;21)
Polymorphonuclear cells
Multipotential progenitors
Common myeloid progenitors
Granulocyte monocyte progenitors
Megakaryocyte-erythroid progenitors
D
DMSO
ATRA
L
HK3
C
CD34+ from MBP
n-fold rel. to D0
0 3 6 9 12
Day of G-CSF differentiation
100kD
total protein
F
HK2-HiBiT
HK3-HiBiT
DMSO
DMSO
ATRA
ATRA
HiBit
total protein
E
G
GFP positive cells [%]
day
4
13
3
19
MOI 2
MOI 10
GFP-P2A-HK2
untransduced
GFP-P2A-HK3
GFP control

## Slide 2
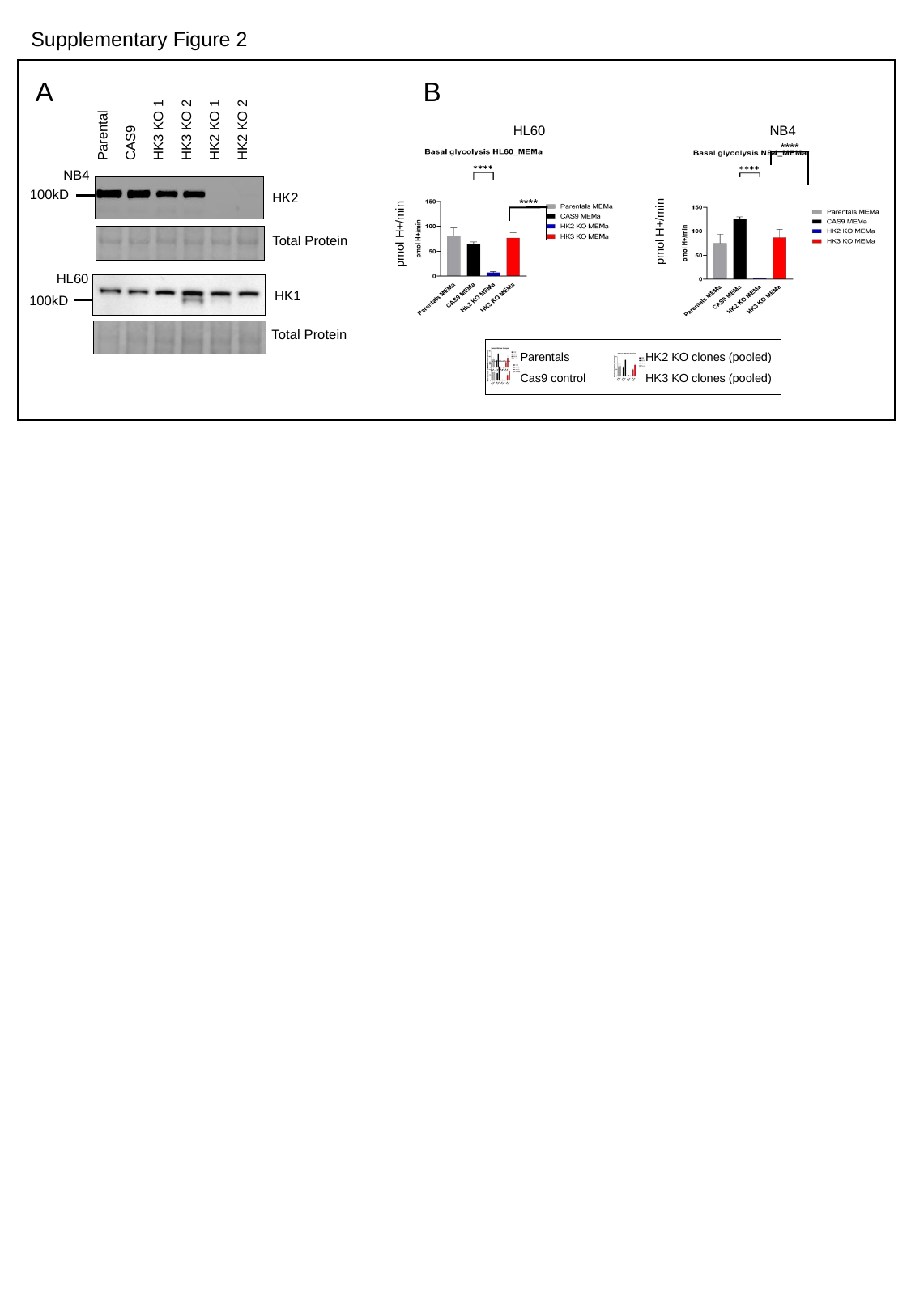

Supplementary Figure 2
B
A
Parental
CAS9
HK3 KO 1
HK3 KO 2
HK2 KO 1
HK2 KO 2
HL60
NB4
****
NB4
100kD
HK2
****
pmol H+/min
pmol H+/min
Total Protein
HL60
HK1
100kD
Total Protein
Parentals
Cas9 control
HK2 KO clones (pooled)
HK3 KO clones (pooled)

## Slide 3
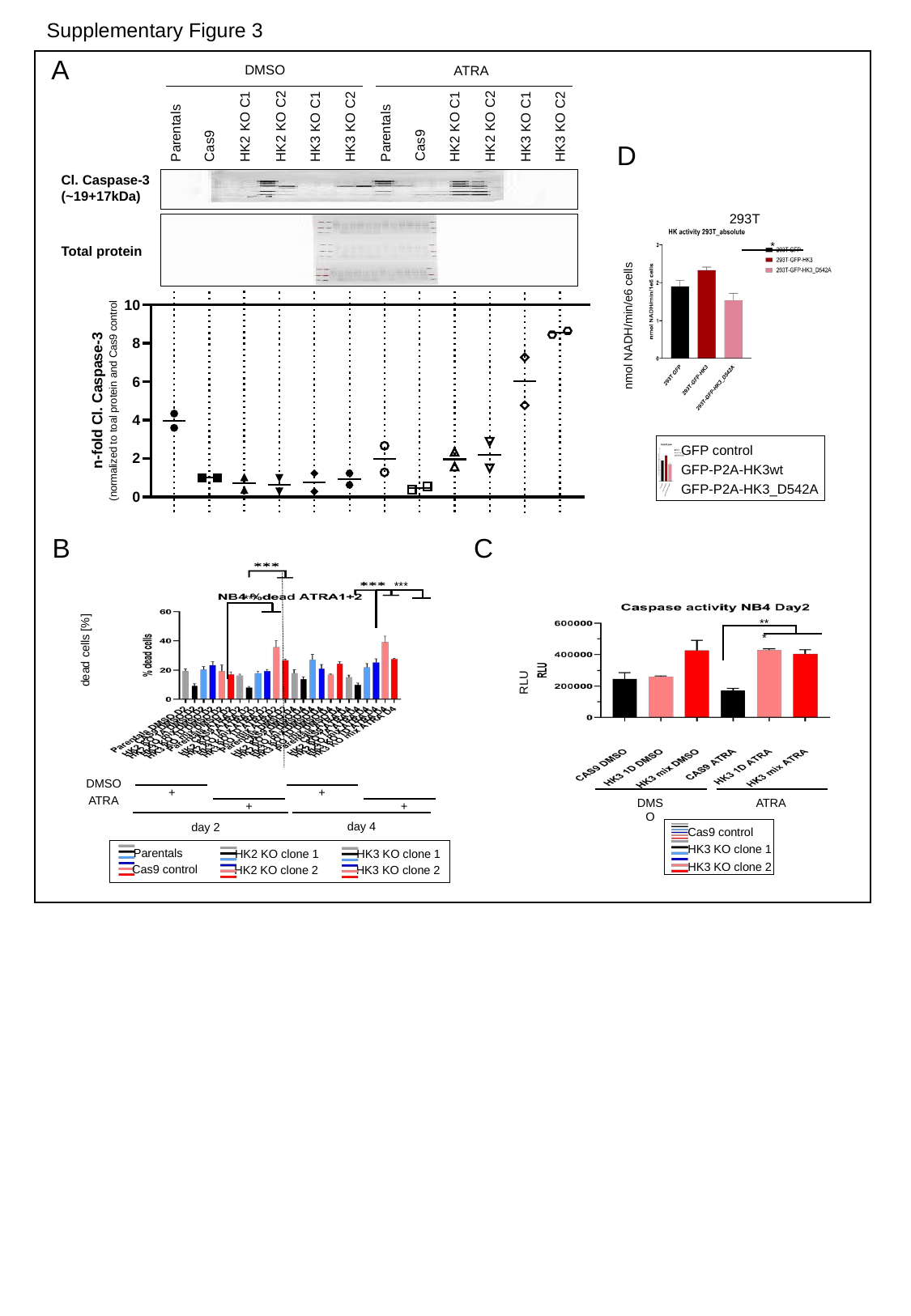

DMSO
ATRA
Cas9
Cas9
HK2 KO C2
HK2 KO C2
Parentals
Parentals
HK2 KO C1
HK3 KO C1
HK3 KO C2
HK2 KO C1
HK3 KO C1
HK3 KO C2
Supplementary Figure 3
A
D
Cl. Caspase-3
(~19+17kDa)
293T
*
Total protein
nmol NADH/min/e6 cells
GFP control
GFP-P2A-HK3wt
GFP-P2A-HK3_D542A
B
C
***
***
***
RLU
DMSO
ATRA
Cas9 control
HK3 KO clone 1
HK3 KO clone 2
dead cells [%]
| DMSO |
| --- |
| ATRA |
+
+
+
+
day 4
day 2
Parentals
HK2 KO clone 1
HK3 KO clone 1
Cas9 control
HK2 KO clone 2
HK3 KO clone 2

## Slide 4
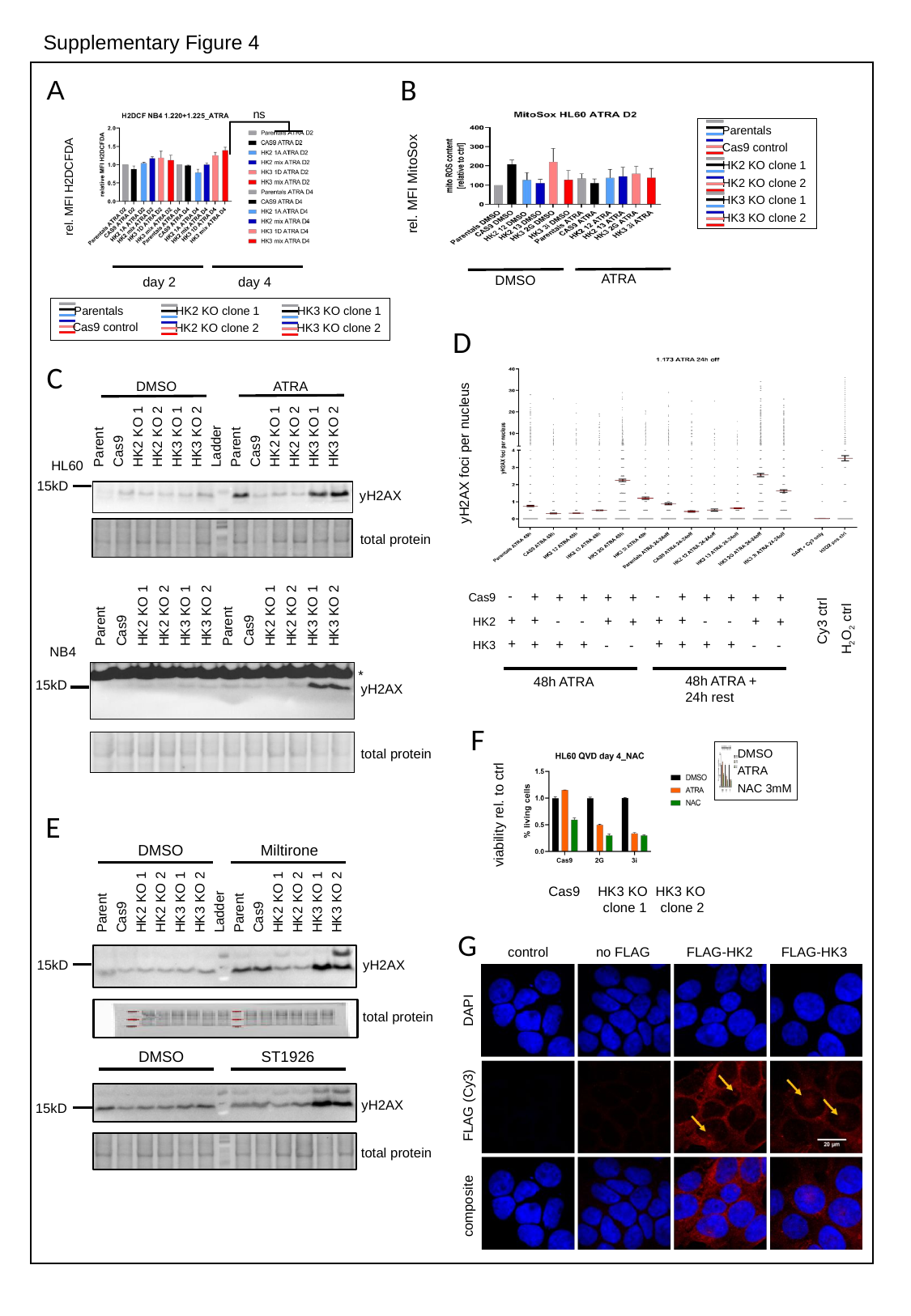

Supplementary Figure 4
B
A
rel. MFI MitoSox
ATRA
DMSO
Parentals
Cas9 control
HK2 KO clone 1
HK2 KO clone 2
HK3 KO clone 1
HK3 KO clone 2
ns
rel. MFI H2DCFDA
day 2
day 4
Parentals
HK2 KO clone 1
HK3 KO clone 1
Cas9 control
HK2 KO clone 2
HK3 KO clone 2
D
yH2AX foci per nucleus
C
DMSO
ATRA
Parent
Cas9
HK2 KO 1
HK2 KO 2
HK3 KO 1
HK3 KO 2
Ladder
Parent
Cas9
HK2 KO 1
HK2 KO 2
HK3 KO 1
HK3 KO 2
HL60
15kD
yH2AX
total protein
Parent
Cas9
HK2 KO 1
HK2 KO 2
HK3 KO 1
HK3 KO 2
Parent
Cas9
HK2 KO 1
HK2 KO 2
HK3 KO 1
HK3 KO 2
Cy3 ctrl
H2O2 ctrl
-
-
+
+
+
+
+
+
+
+
+
+
| Cas9 |
| --- |
| HK2 |
| HK3 |
+
+
+
+
-
-
-
-
+
+
+
+
+
+
+
+
+
+
+
+
-
-
-
-
NB4
*
15kD
yH2AX
total protein
48h ATRA + 24h rest
48h ATRA
F
DMSO
ATRA
NAC 3mM
viability rel. to ctrl
E
DMSO
Miltirone
Parent
Cas9
HK2 KO 1
HK2 KO 2
HK3 KO 1
HK3 KO 2
Ladder
Parent
Cas9
HK2 KO 1
HK2 KO 2
HK3 KO 1
HK3 KO 2
Cas9
HK3 KO
clone 1
HK3 KO
clone 2
G
control
no FLAG
FLAG-HK2
FLAG-HK3
15kD
yH2AX
DAPI
total protein
DMSO
ST1926
yH2AX
15kD
total protein
FLAG (Cy3)
composite

## Slide 5
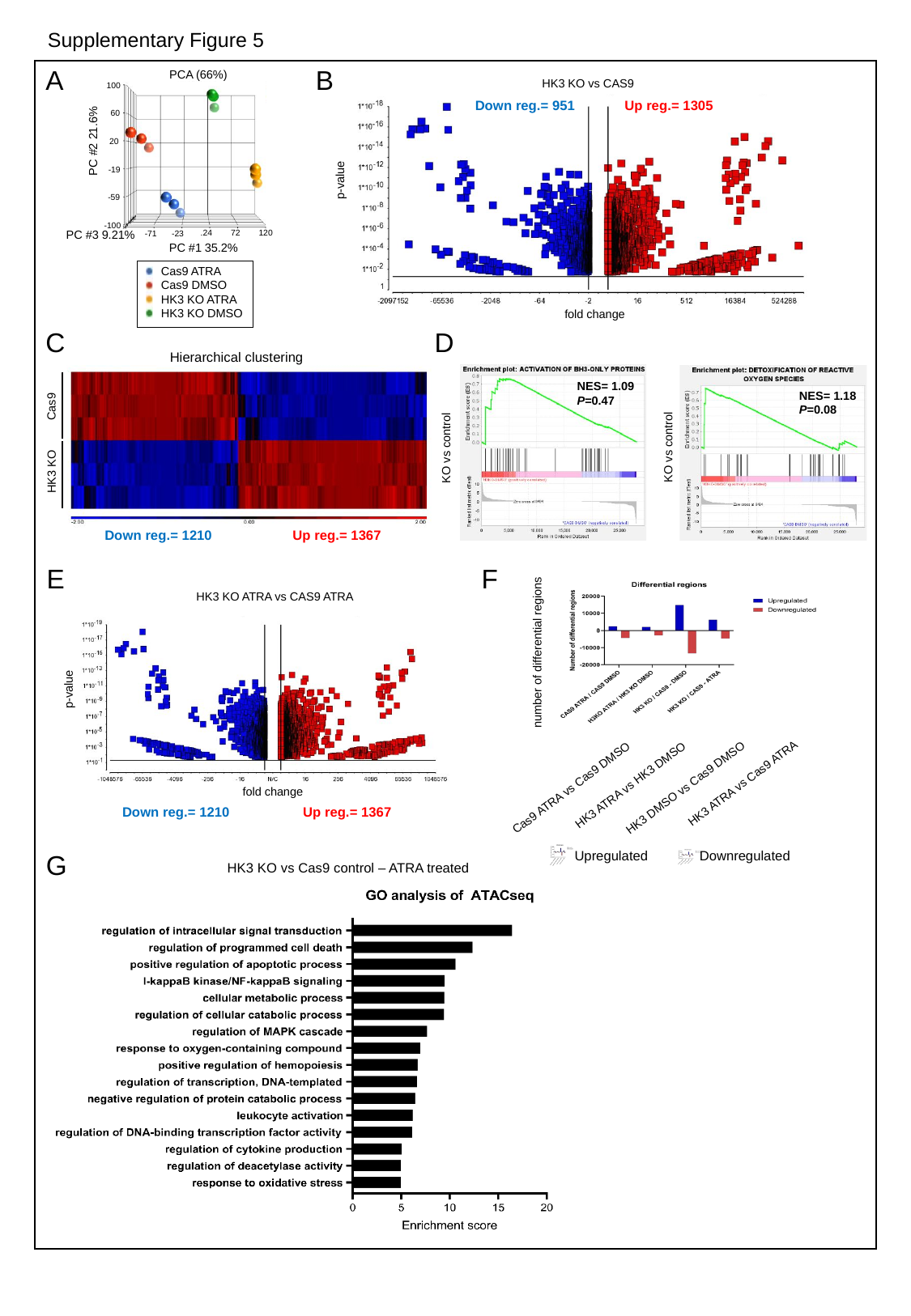

Supplementary Figure 5
A
B
PCA (66%)
HK3 KO vs CAS9
100
Down reg.= 951
Up reg.= 1305
p-value
fold change
60
PC #2 21.6%
20
-19
-59
-100
PC #3 9.21%
.24
72
120
-71
-23
PC #1 35.2%
Cas9 ATRA
Cas9 DMSO
HK3 KO ATRA
HK3 KO DMSO
C
D
Hierarchical clustering
Cas9
HK3 KO
Down reg.= 1210
Up reg.= 1367
NES= 1.09
P=0.47
NES= 1.18
P=0.08
KO vs control
KO vs control
E
F
number of differential regions
HK3 ATRA vs Cas9 ATRA
HK3 DMSO vs Cas9 DMSO
HK3 ATRA vs HK3 DMSO
Downregulated
Upregulated
Cas9 ATRA vs Cas9 DMSO
HK3 KO ATRA vs CAS9 ATRA
p-value
fold change
Down reg.= 1210
Up reg.= 1367
G
HK3 KO vs Cas9 control – ATRA treated

## Slide 6
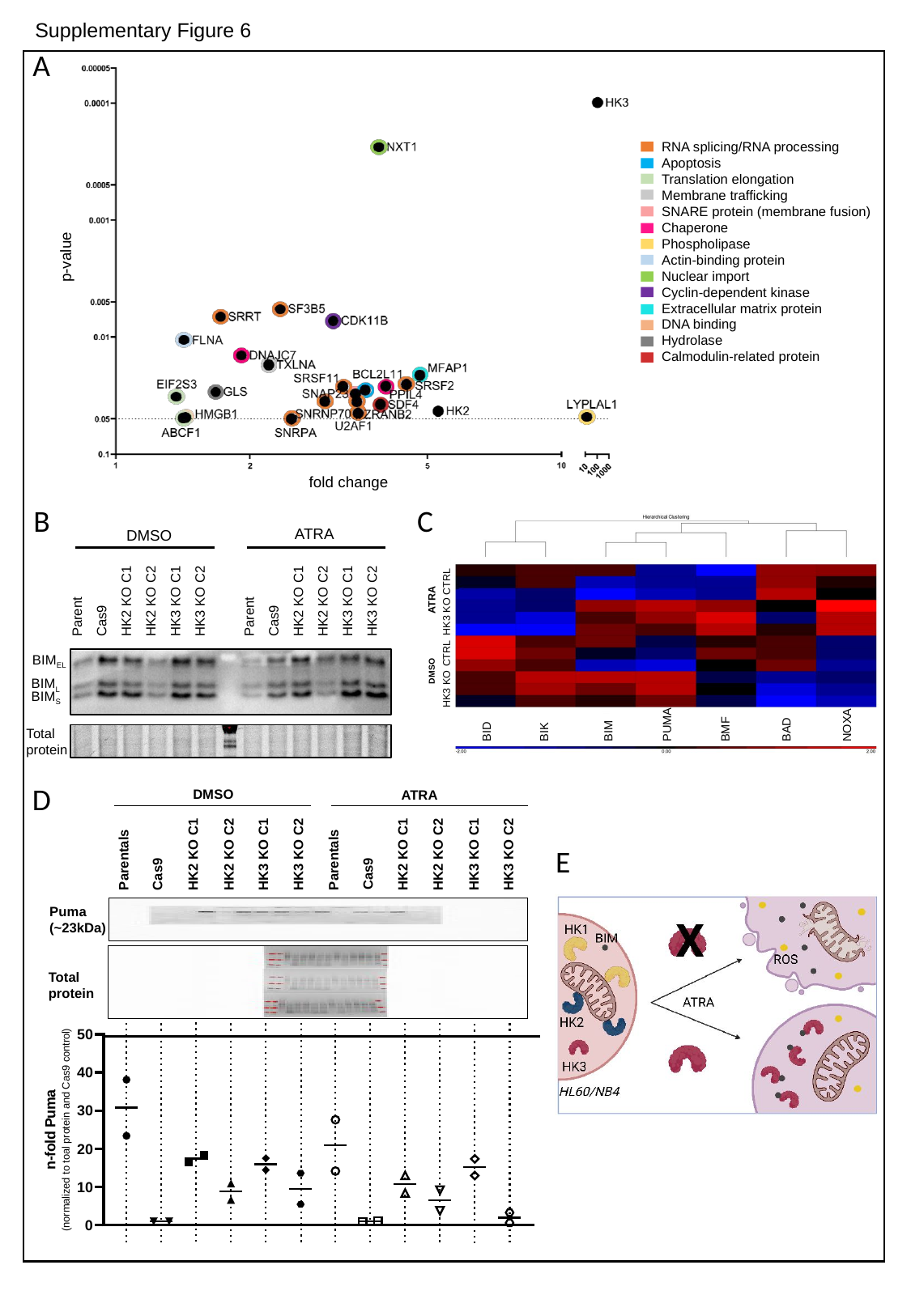

Supplementary Figure 6
A
RNA splicing/RNA processing
Apoptosis
Translation elongation
Membrane trafficking
SNARE protein (membrane fusion)
Chaperone
Phospholipase
Actin-binding protein
Nuclear import
Cyclin-dependent kinase
Extracellular matrix protein
DNA binding
Hydrolase
Calmodulin-related protein
p-value
fold change
B
C
ATRA
DMSO
Parent
Cas9
HK2 KO C1
HK2 KO C2
HK3 KO C1
HK3 KO C2
Parent
Cas9
HK2 KO C1
HK2 KO C2
HK3 KO C1
HK3 KO C2
CTRL
ATRA
HK3 KO
CTRL
BIMEL
DMSO
BIML
HK3 KO
BIMS
BID
BIK
BIM
PUMA
BMF
BAD
NOXA
DMSO
ATRA
Cas9
Cas9
HK2 KO C2
HK2 KO C2
Parentals
Parentals
HK2 KO C1
HK3 KO C1
HK3 KO C2
HK2 KO C1
HK3 KO C1
HK3 KO C2
Total
protein
D
E
Puma
(~23kDa)
Total
protein
