# Supplementary material for: Hexokinase 3 enhances myeloid cell survival via non-glycolytic functions
Source: Cell Death Dis. 2022 May 11;13(5):448. doi: 10.1038/s41419-022-04891-w (PMC9091226; doi:10.1038/s41419-022-04891-w)
Supplement: Supplementary file 4 — Supplementary Information [file 41419_2022_4891_MOESM4_ESM.docx]

**Supplementary Information**

**Hexokinase 3 enhances myeloid cell survival via non-glycolytic functions**

Kristina Seiler^1,2,3^, Magali Humbert^1^, Petra Minder^3^, Iris Mashimo^3^, Deborah Krauer^1^, Elena Federzoni^3^, Bich Vu^4^, James J. Moresco^5^, John R. Yates III^5^, Martin C. Sadowski^1^, Ramin Radpour^6,7^, Thomas Kaufmann^8^, Jean-Emmanuel Sarry^9,10^, Joern Dengjel^4^, Mario P. Tschan^1,2,*^ and Bruce E. Torbett^3,11,12,13*^

**Supplementary methods**

**RNA-seq and ATAC-seq data analyses**

The reads generated by RNA-Seq were mapped to the human reference genome (GRCh38/hg38). The level of gene expression was assessed after RPKM normalization and log2 transformation and genes with mean values a log2 RPKM>3 for at least one of the conditions, were kept in the analysis. The data set was analyzed by two-way ANOVA. After statistical analysis, genes with significant difference in their expression at *p*-value<0.05 and fold differences ≥1.5 were selected. Principal component analysis (PCA) analysis was used to map the variations among profiled samples. Data were clustered using standard Euclidean’s method based on the average linkage and heatmaps were generated according to the standard normal distribution of the values.

The paired-end 42 bp sequencing reads (PE42) generated by Illumina sequencing (using NextSeq 500) were mapped to the genome using the BWA algorithm with default settings (“bwa mem”). Alignment information for each read was stored in the BAM format. Only reads that pass Illumina’s purity filter, align with no more than 2 mismatches, and map uniquely to the genome were used in the subsequent analysis. In addition, duplicate reads (“PCR duplicates”) were removed. Genomic regions with high levels of transposition/tagging events were determined using the MACS2 peak calling algorithm^1^. Since both reads (tags) from paired-end sequencing represent transposition events, both reads were used for peak-calling but treated as single, independent reads. To identify the density of transposition events along the genome, the genome was divided into 32 bp bins and the number of fragments in each bin was determined. For this purpose, reads were extended to 200 bp, which is close to the average length of the sequenced library inserts. This information (“signal map”; histogram of fragment densities) was stored in bigWig files, which are also the basis of the peak metrics in the Active Motif analysis program. Normalization: In the default analysis, the tag number of all samples was reduced (by random sampling) to the number of tags present in the smallest sample. To compare peak metrics between 2 or more samples, overlapping Intervals were grouped into “Merged Regions”, which are defined by the start coordinate of the most upstream Interval and the end coordinate of the most downstream Interval (= union of overlapping Intervals; “merged peaks”). In locations where only one sample has an Interval, this Interval defines the Merged Region. The use of Merged Regions is necessary because the locations and lengths of Intervals are rarely exactly the same when comparing different samples. Furthermore, with this approach fragment density values can be obtained even for samples for which no peak was called. After defining the Intervals and Merged Regions, their genomic locations along with their proximities to gene annotations and other genomic features were determined. In addition, average and peak (i.e. at “summit”) fragment densities within Intervals and Merged Regions were compiled.

The OMICs data compiled for this study were made publicly available on the Gene Expression Omnibus (GEO) website (http://www.ncbi.nlm.nih.gov/geo/) under the accession number GSE197164.

1. Zhang, Y., Liu, T., Meyer, C.A. et al. Model-based Analysis of ChIP-Seq (MACS). Genome Biol **9**, R137 (2008).
