# Supplementary material for: Hexokinase 3 enhances myeloid cell survival via non-glycolytic functions
Source: Cell Death Dis. 2022 May 11;13(5):448. doi: 10.1038/s41419-022-04891-w (PMC9091226; doi:10.1038/s41419-022-04891-w)
Supplement: Supplementary file 5 — Original western blots [file 41419_2022_4891_MOESM5_ESM.pptx]

## Slide 1
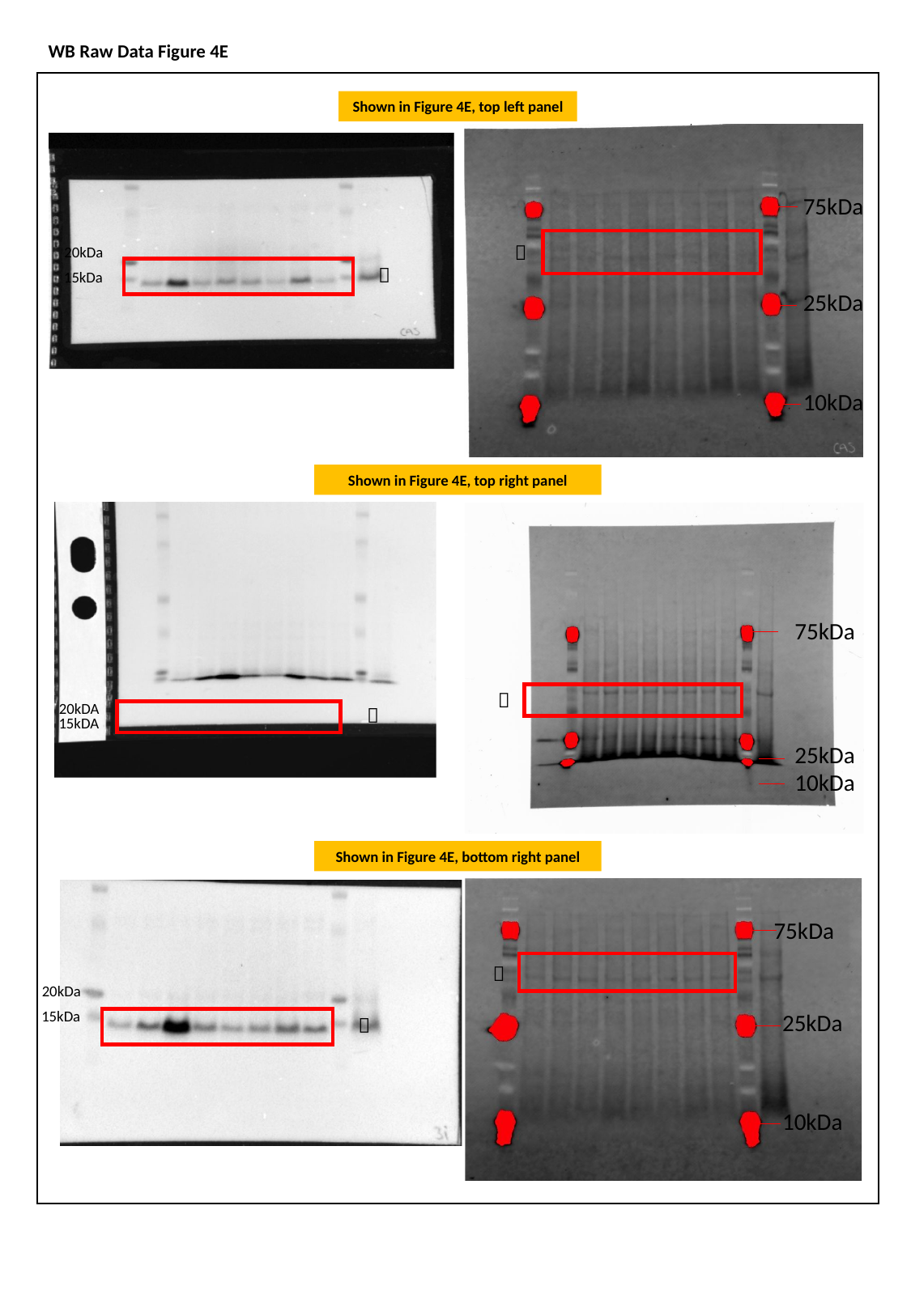

WB Raw Data Figure 4E
Shown in Figure 4E, top left panel
75kDa

20kDa

15kDa
25kDa
10kDa
Shown in Figure 4E, top right panel
75kDa

20kDA

15kDA
25kDa
10kDa
Shown in Figure 4E, bottom right panel
75kDa

20kDa
15kDa
25kDa

10kDa

## Slide 2
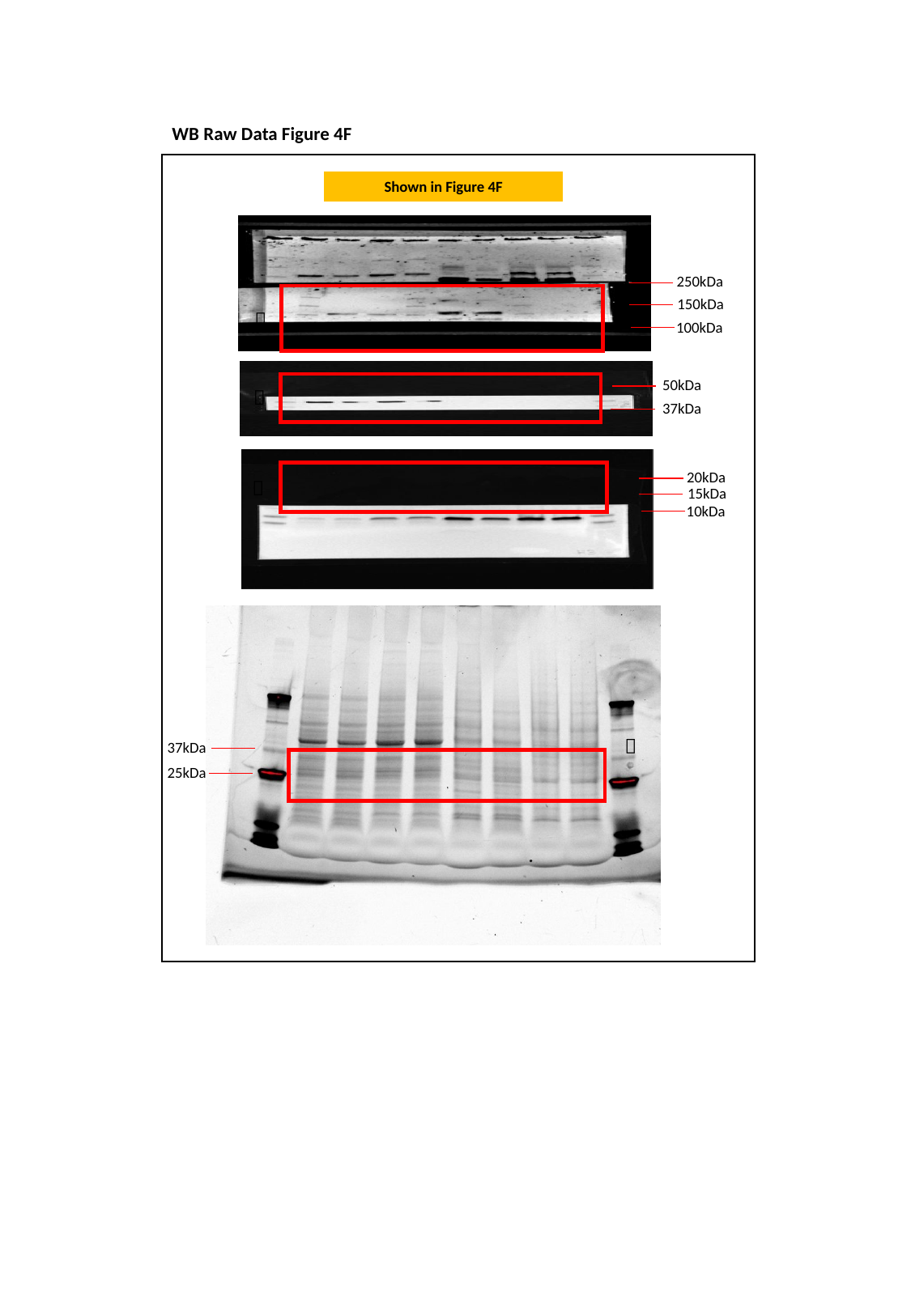

WB Raw Data Figure 4F
Shown in Figure 4F
250kDa
150kDa

100kDa
50kDa

37kDa
20kDa

15kDa
10kDa

37kDa
25kDa

## Slide 3
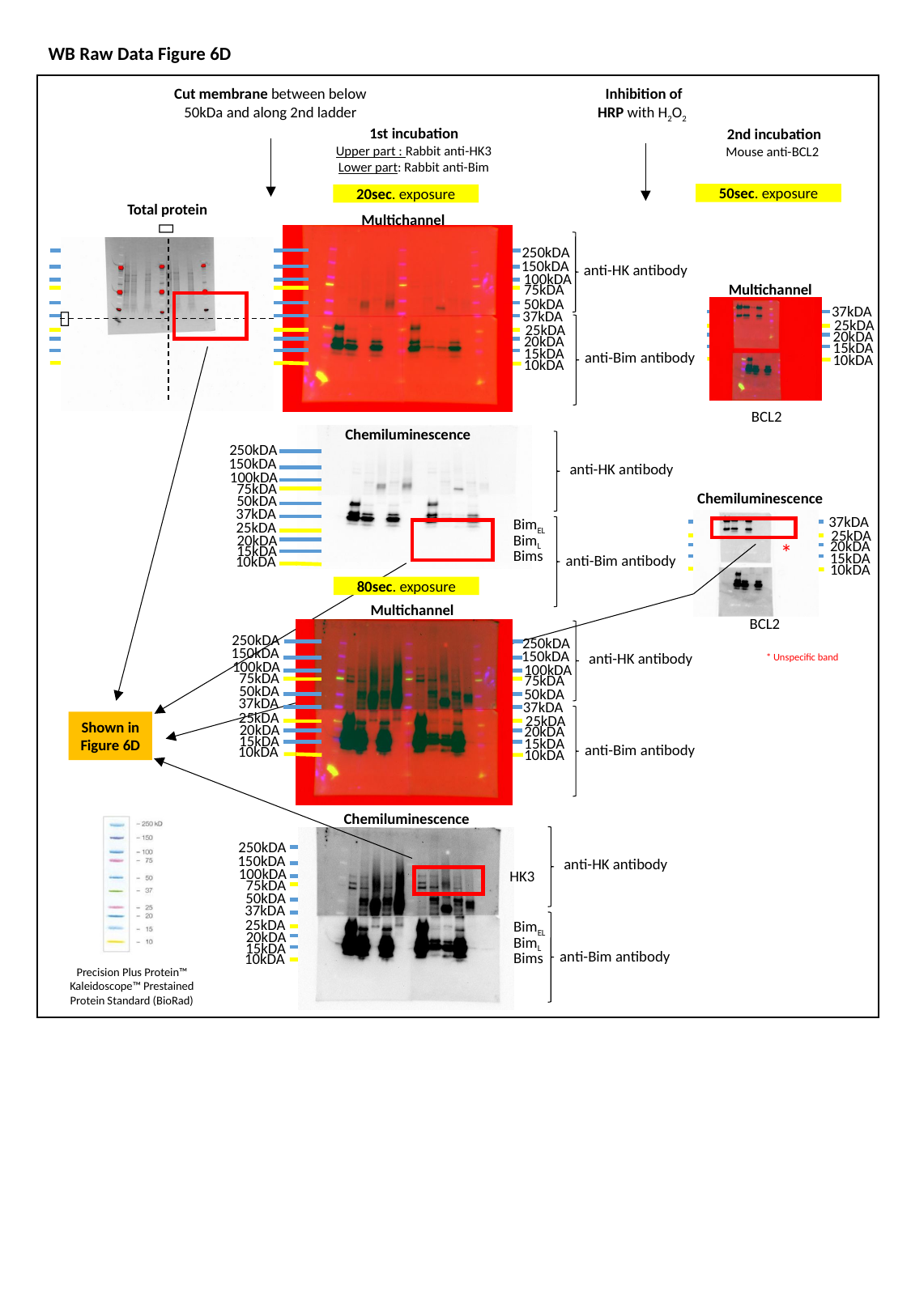

WB Raw Data Figure 6D
Cut membrane between below 50kDa and along 2nd ladder
Inhibition of HRP with H2O2
1st incubation
Upper part : Rabbit anti-HK3
Lower part: Rabbit anti-Bim
2nd incubation
Mouse anti-BCL2
50sec. exposure
20sec. exposure
Total protein
Multichannel

250kDA
150kDA
100kDA
75kDA
50kDA
37kDA
25kDA
20kDA
15kDA
10kDA
anti-HK antibody
Multichannel
37kDA
25kDA
20kDA
15kDA
10kDA

anti-Bim antibody
BCL2
Chemiluminescence
250kDA
150kDA
100kDA
75kDA
50kDA
37kDA
25kDA
20kDA
15kDA
10kDA
anti-HK antibody
Chemiluminescence
37kDA
25kDA
20kDA
15kDA
10kDA
BimEL
BimL
*
Bims
anti-Bim antibody
80sec. exposure
Multichannel
BCL2
250kDA
150kDA
100kDA
75kDA
50kDA
37kDA
25kDA
20kDA
15kDA
10kDA
250kDA
150kDA
100kDA
75kDA
50kDA
37kDA
25kDA
20kDA
15kDA
10kDA
anti-HK antibody
* Unspecific band
Shown in
Figure 6D
anti-Bim antibody
Chemiluminescence
250kDA
150kDA
100kDA
75kDA
50kDA
37kDA
25kDA
20kDA
15kDA
10kDA
anti-HK antibody
HK3
BimEL
BimL
anti-Bim antibody
Bims
Precision Plus Protein™ Kaleidoscope™ Prestained Protein Standard (BioRad)

## Slide 4
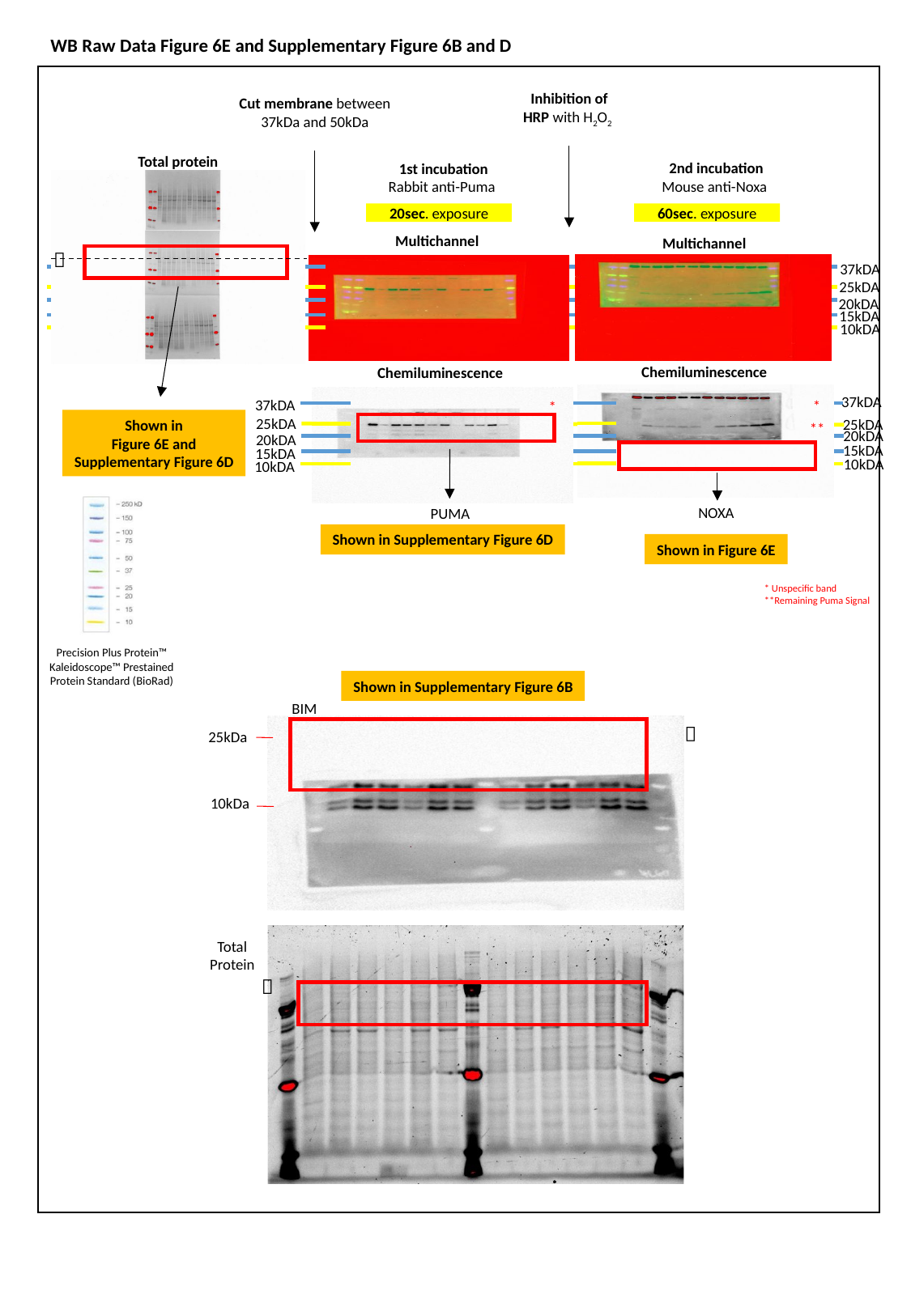

WB Raw Data Figure 6E and Supplementary Figure 6B and D
Inhibition of HRP with H2O2
Cut membrane between 37kDa and 50kDa
Total protein
2nd incubation
Mouse anti-Noxa
1st incubation
Rabbit anti-Puma
60sec. exposure
20sec. exposure
Multichannel
Multichannel

37kDA
25kDA
20kDA
15kDA
10kDA
Chemiluminescence
Chemiluminescence
37kDA
37kDA
*
*
25kDA
25kDA
Shown in
Figure 6E and
Supplementary Figure 6D
**
20kDA
20kDA
15kDA
15kDA
10kDA
10kDA
NOXA
PUMA
Shown in Supplementary Figure 6D
Shown in Figure 6E
* Unspecific band
**Remaining Puma Signal
Precision Plus Protein™ Kaleidoscope™ Prestained Protein Standard (BioRad)
Shown in Supplementary Figure 6B
BIM

25kDa
10kDa
Total Protein


## Slide 5
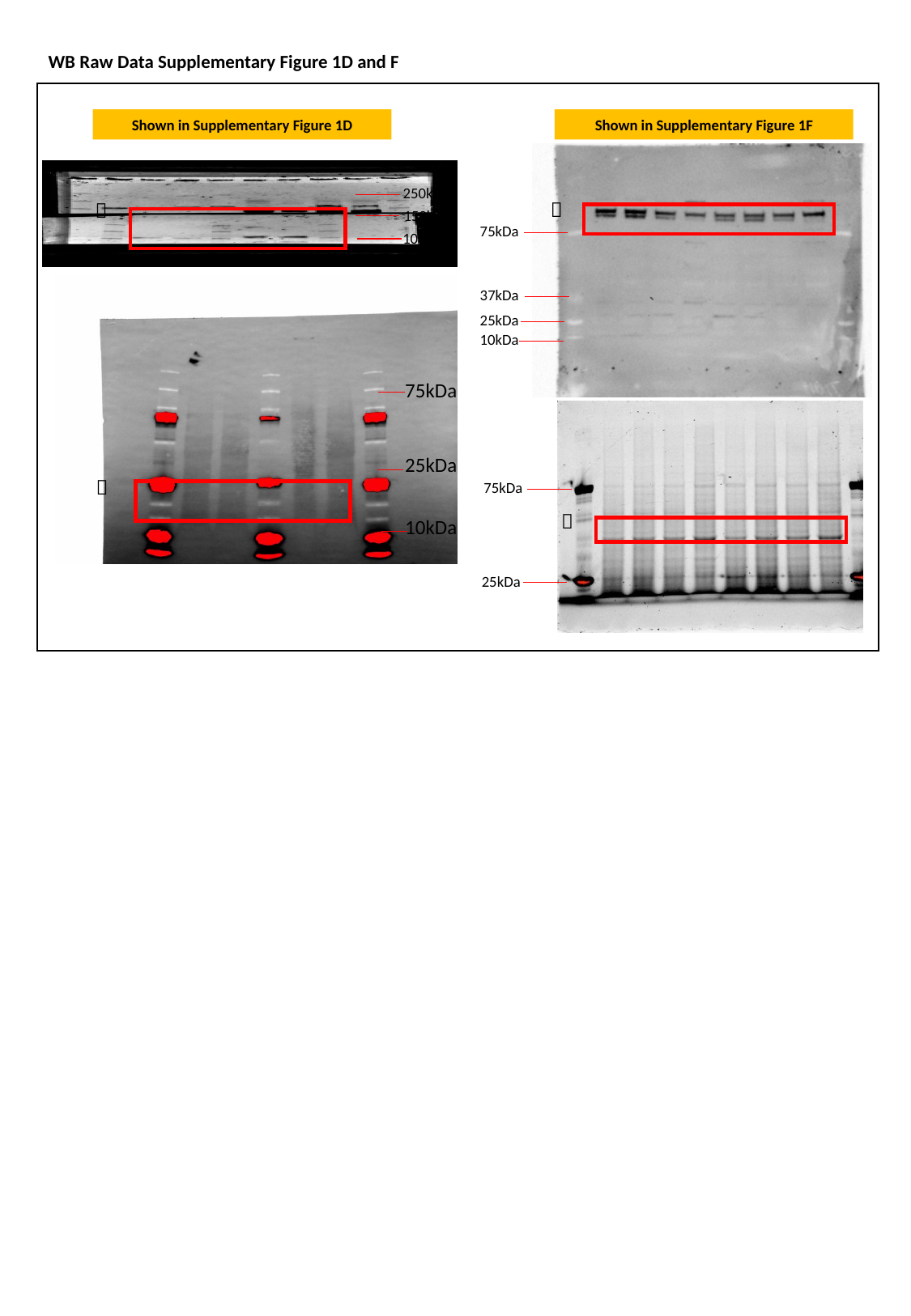

WB Raw Data Supplementary Figure 1D and F
Shown in Supplementary Figure 1D
Shown in Supplementary Figure 1F
250kDa


150kDa
75kDa
100kDa
37kDa
25kDa
10kDa
75kDa
25kDa

75kDa
25kDa

10kDa

## Slide 6
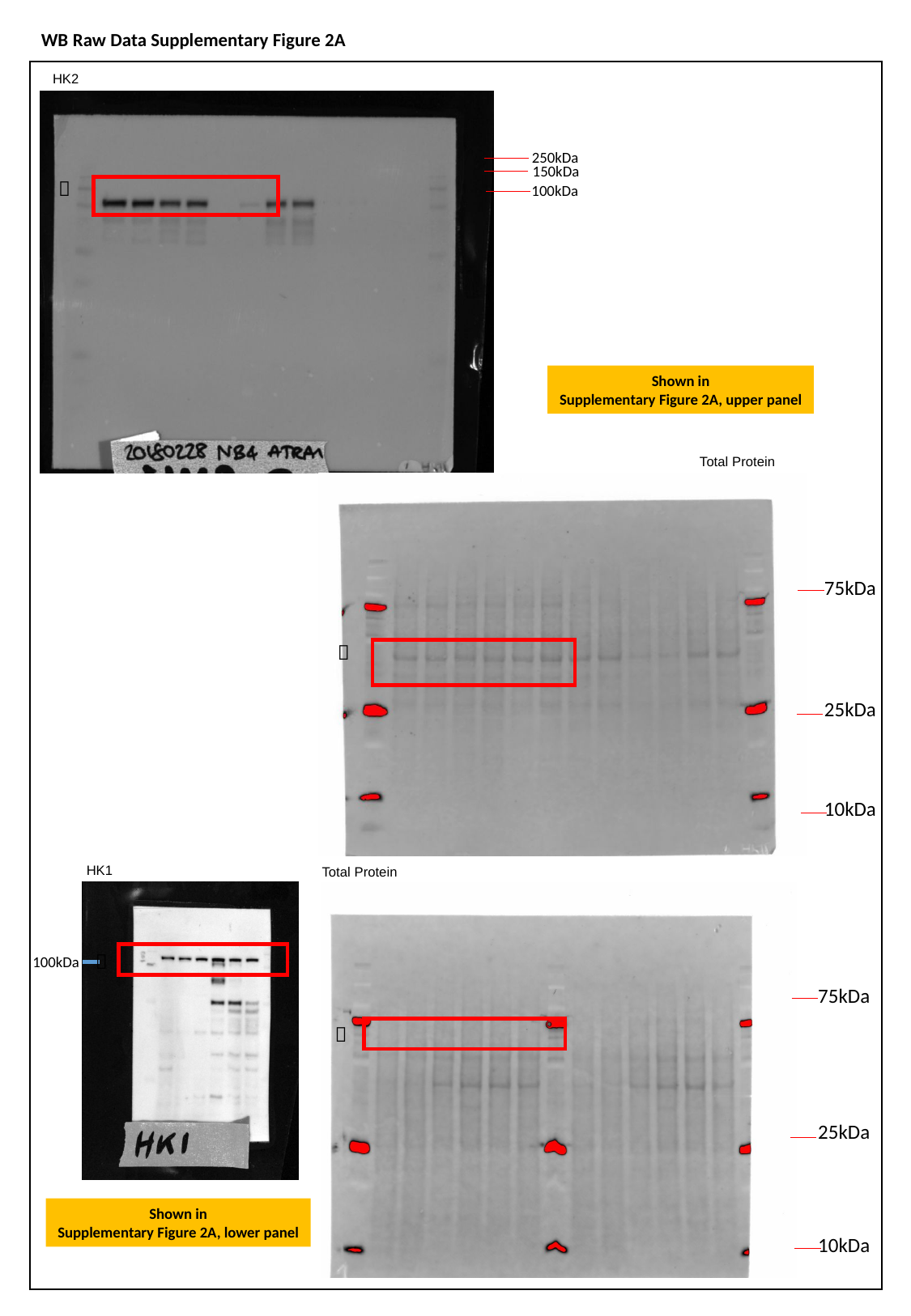

WB Raw Data Supplementary Figure 2A
HK2
250kDa
150kDa

100kDa
Shown in
Supplementary Figure 2A, upper panel
Total Protein
75kDa

25kDa
10kDa
HK1
Total Protein

100kDa
75kDa

25kDa
Shown in
Supplementary Figure 2A, lower panel
10kDa

## Slide 7
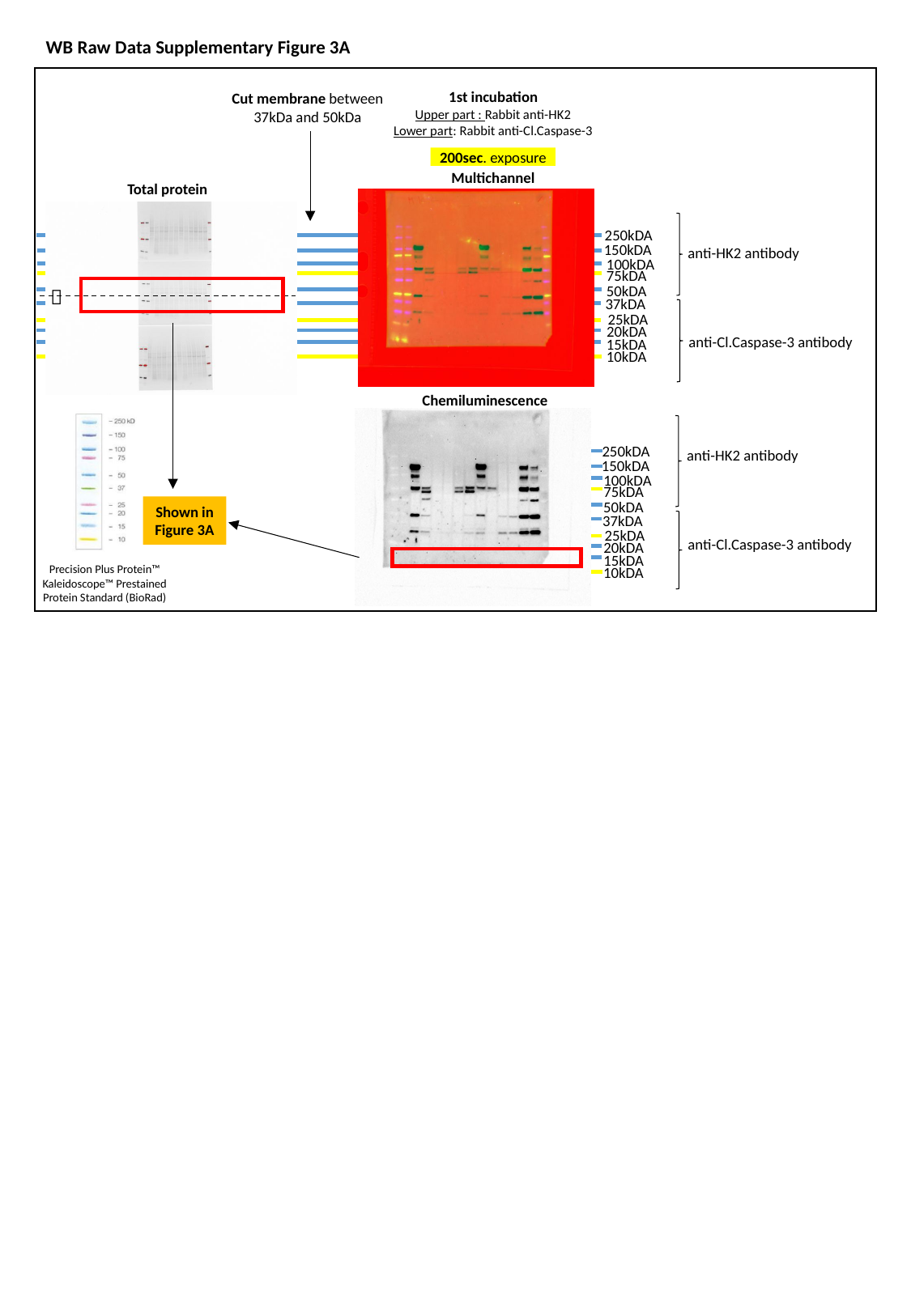

WB Raw Data Supplementary Figure 3A
1st incubation
Upper part : Rabbit anti-HK2
Lower part: Rabbit anti-Cl.Caspase-3
Cut membrane between 37kDa and 50kDa
200sec. exposure
Multichannel
Total protein
1st antibody incubation
Rabbit anti-Puma
250kDA
150kDA
100kDA
75kDA
50kDA
37kDA
25kDA
20kDA
15kDA
10kDA
20sec. exposure
anti-HK2 antibody
Multichannel

anti-Cl.Caspase-3 antibody
Chemiluminescence
250kDA
150kDA
100kDA
75kDA
50kDA
37kDA
25kDA
20kDA
15kDA
10kDA
anti-HK2 antibody
Shown in
Figure 3A
PUMA
anti-Cl.Caspase-3 antibody
Shown in Supplementary Figure 6E
Precision Plus Protein™ Kaleidoscope™ Prestained Protein Standard (BioRad)

## Slide 8
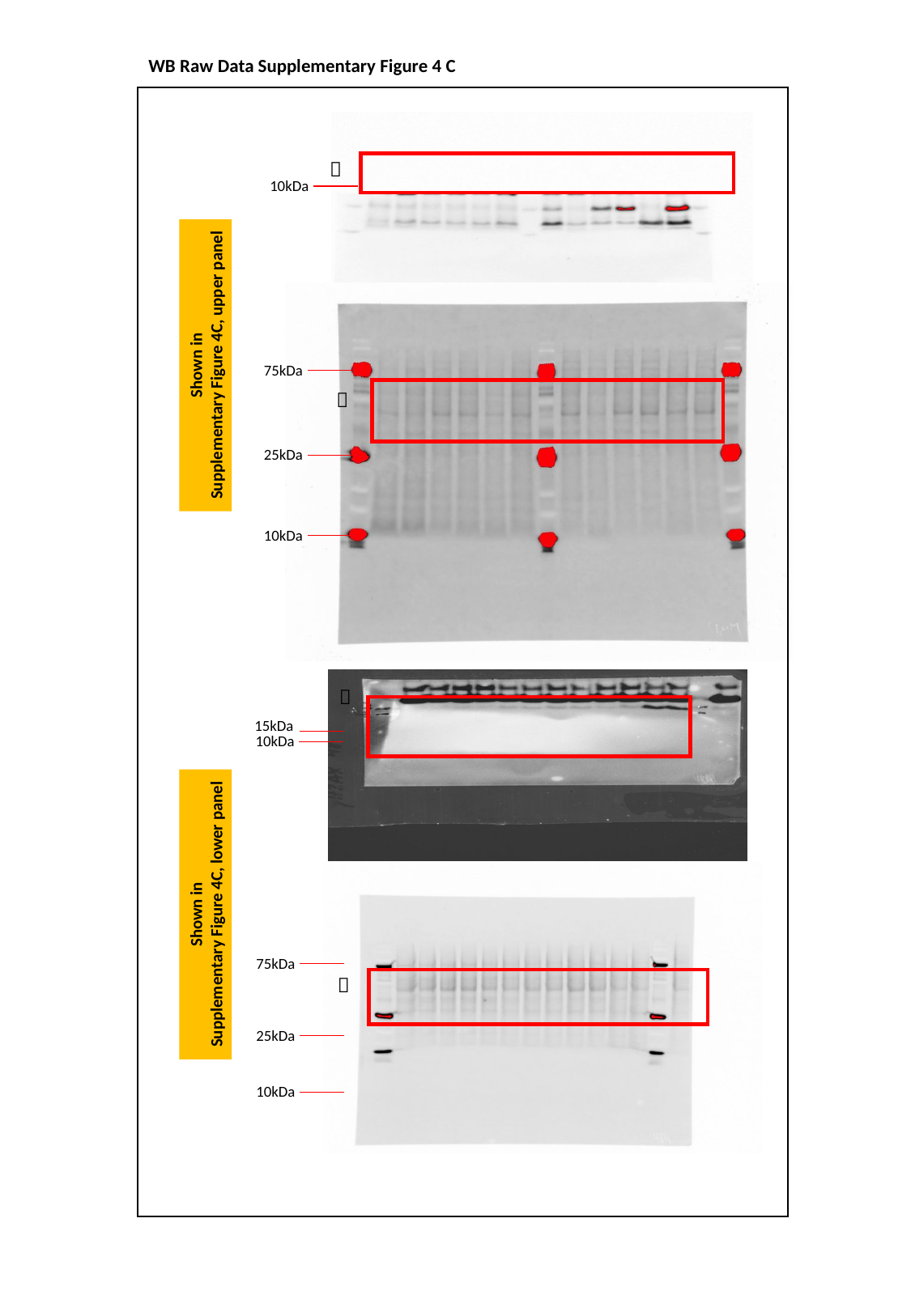

WB Raw Data Supplementary Figure 4 C

10kDa
Shown in
Supplementary Figure 4C, upper panel
75kDa

25kDa
10kDa

15kDa
10kDa
Shown in
Supplementary Figure 4C, lower panel
75kDa

25kDa
10kDa

## Slide 9
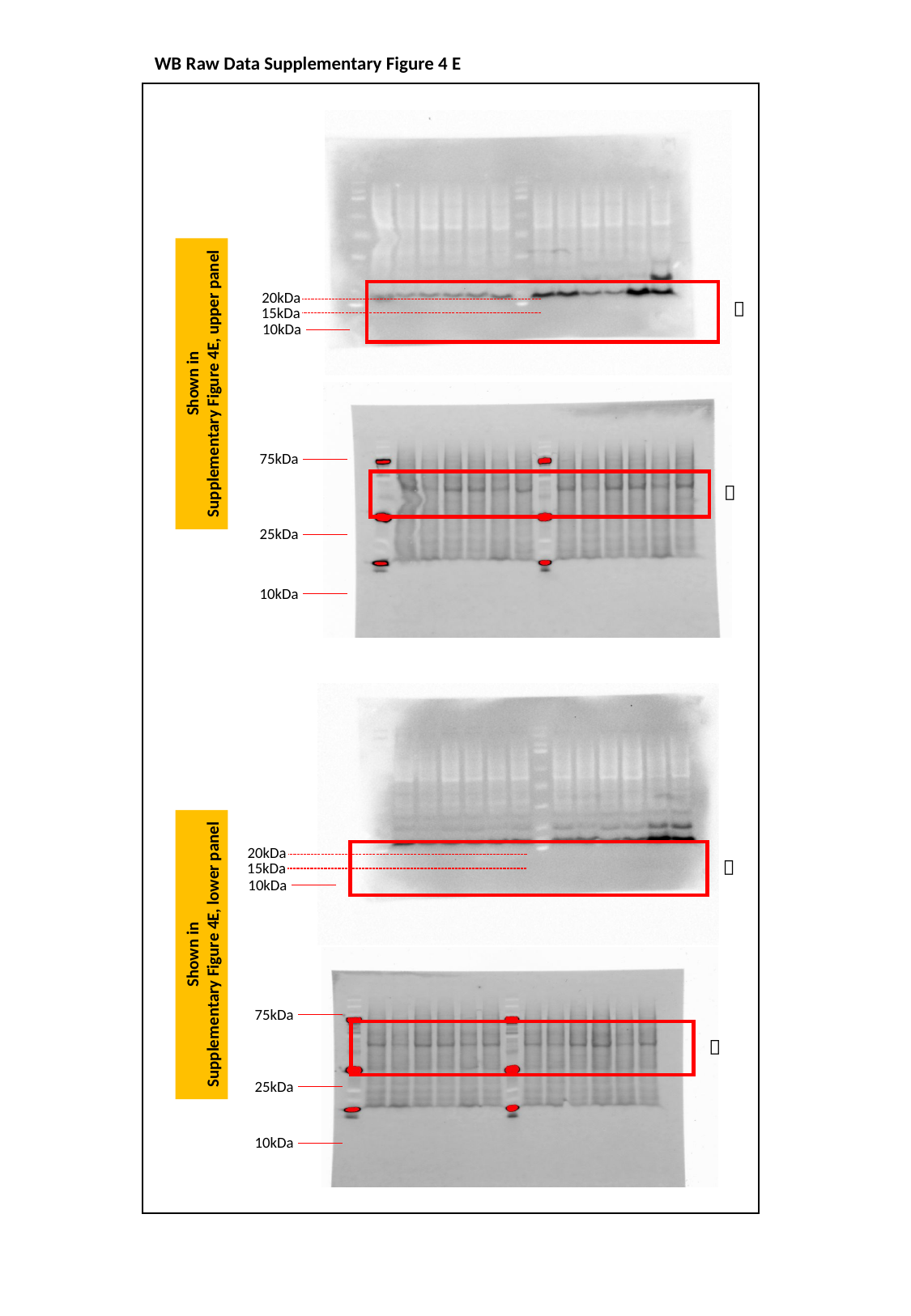

WB Raw Data Supplementary Figure 4 E
20kDa

15kDa
10kDa
Shown in
Supplementary Figure 4E, upper panel
75kDa

25kDa
10kDa
20kDa

15kDa
10kDa
Shown in
Supplementary Figure 4E, lower panel
75kDa

25kDa
10kDa
