# Supplementary material for: Hexokinase 3 enhances myeloid cell survival via non-glycolytic functions
Source: Cell Death Dis. 2022 May 11;13(5):448. doi: 10.1038/s41419-022-04891-w (PMC9091226; doi:10.1038/s41419-022-04891-w)
Supplement: Supplementary file 7 — Agreement co-authors [file 41419_2022_4891_MOESM7_ESM.pdf]

**From:** **Bill, Anna Magdalena (PATHOLOGY)** anna.schlaefli@pathology.unibe.ch
**Subject:** AW: important: PLEASE CONFIRM author list CDDIS-21-2729RR
**Date:** 12 April 2022 at 20:14
**To:** Tschan, Mario (PATHOLOGY) mario.tschan@pathology.unibe.ch

Dear Mario
I agree to be a co-author on this paper.
Best wishes
Anna Schläfli

------ Originalnachricht------
**Von:** Tschan, Mario (PATHOLOGY)
**Datum:** Di., 12. Apr. 2022 20:40
**An:** Seiler, Kristina (PATHOLOGY);Magali Humbert;petra.minder@gmx.ch;mimashimo@gmail.com;Bill, Anna Magdalena (PATHOLOGY);Krauer, Deborah (PATHOLOGY);EA F;bich.vu@unifr.ch;jmoresco@scripps.edu;jyates@scripps.edu;Sadowski, Martin (PATHOLOGY);Radpour, Ramin (DBMR);Kaufmann, Thomas (PKI);Jean-emmanuel Sarry;Jörn Dengjel;Tschan, Mario (PATHOLOGY);Bruce E Torbett;
**Cc:**
**Betreff:**important: PLEASE CONFIRM author list CDDIS-21-2729RR

Dear colleagues,
Extensive revision experiments done by Anna M. Schläfli qualified her as a co-author on our paper. CDDis requires that all authors agree on the new author list (see below and attachment).

"Please request agreement from all authors including additions and deletions, these can be collected in the following way:

Email your co-authors with the change, and ask them to reply to your email confirming that they agree to these changes. Once you have collected these replies, please combine all of the co-authors' email responses in one document and upload this file to your submission."

PLEASE REPLY TO THIS MAIL AND LET ME KNOW IF YOU AGREE. *Thank you.*

!! The manuscript submission can only continue once all authors replied !!

All the best,

Mario

**From:** **Bruce E Torbett** betorbet@uw.edu
**Subject:** Re: important: PLEASE CONFIRM author list CDDIS-21-2729RR
**Date:** 12 April 2022 at 19:58
**To:** mario.tschan@pathology.unibe.ch
**Cc:** kristina.seiler@pathology.unibe.ch, magali.humbert@yahoo.fr, petra.minder@gmx.ch, mimashimo@gmail.com, anna.schlaefli@pathology.unibe.ch, deborah.shan@pathology.unibe.ch, federzoni.elena@gmail.com, bich.vu@unifr.ch, Jean-emmanuel Sarry jean-emmanuel.sarry@inserm.fr, jmoresco@scripps.edu, jyates@scripps.edu, martin.sadowski@pathology.unibe.ch, ramin.radpour@dbmr.unibe.ch, thomas.kaufmann@pki.unibe.ch, joern.dengjel@unifr.ch, Bruce E Torbett betorbet@uw.edu

---

Hi Mario – I strongly support Anna M. Schläfli be included as an author on the resubmission.

Best,

Bruce

---------------------------------------------------------------------------------

***Bruce E. Torbett, PhD, MSPH***
Professor of Pediatrics,
School of Medicine,
University of Washington
And Seattle Children's Research Institute (SCRI)
Associate Director, Center for Immunity and Immunotherapies @ SCRI
Institute for Stem Cell and Regenerative Medicine
Co-Director, HIV interaction in Viral Evolution (HIVE) Center

Email: betorbet@uw.edu
Phone: 206-884-1140
FAX: 206-987-7310
Cell: 760-518-6730

Torbett lab: https://torbettlab.org
HIVE Center: http://HIVEcenter.net

**Mailing and All FEDEX or Deliveries Use:**
Bruce Torbett, PhD, MSPH
Seattle Children's Research Institute
M/S JMB-7
1900 Ninth Avenue
Seattle, WA 98101-1309

----------------------------------------------------------------------------------------------------------------------------------------------------------------------------------------------------

SECURITY/CONFIDENTIALITY WARNING: This message and any attachments are intended solely for the individual or entity to which they are addressed. This communication may contain information that is privileged, confidential, or exempt from disclosure under applicable law. Since this e-mail has been sent without encryption, individuals other than the intended recipient may be able to view the information, forward it to others, or in some way tamper with the information without the knowledge or consent of the sender. If you are not the intended recipient, or the employee or person responsible for delivering [illegible]

**From:** Krauer, Deborah **(PATHOLOGY)** deborah.shan@pathology.unibe.ch
**Subject:** AW: important: PLEASE CONFIRM author list CDDIS-21-2729RR
**Date:** 13 April 2022 at 07:36
**To:** Tschan, Mario (PATHOLOGY) mario.tschan@pathology.unibe.ch

Dear Mario

I agree that Anna M.Schläfli is co-author.

Best
Debby

---

**Von:** Tschan, Mario (PATHOLOGY) <mario.tschan@pathology.unibe.ch>
**Gesendet:** Dienstag, 12. April 2022 20:41
**An:** Seiler, Kristina (PATHOLOGY) <kristina.seiler@pathology.unibe.ch>; Magali Humbert <magali.humbert@yahoo.fr>; petra.minder@gmx.ch; mimashimo@gmail.com; Bill, Anna Magdalena (PATHOLOGY) <anna.schlaefli@pathology.unibe.ch>; Krauer, Deborah (PATHOLOGY) <deborah.shan@pathology.unibe.ch>; EA F <federzoni.elena@gmail.com>; bich.vu@unifr.ch; jmoresco@scripps.edu; jyates@scripps.edu; Sadowski, Martin (PATHOLOGY) <martin.sadowski@pathology.unibe.ch>; Radpour, Ramin (DBMR) <ramin.radpour@dbmr.unibe.ch>; Kaufmann, Thomas (PKI) <thomas.kaufmann@pki.unibe.ch>; Jean-emmanuel Sarry <jean-emmanuel.sarry@inserm.fr>; Jörn Dengjel <joern.dengjel@unifr.ch>; Tschan, Mario (PATHOLOGY) <mario.tschan@pathology.unibe.ch>; Bruce E Torbett <betorbet@uw.edu>
**Betreff:** important: PLEASE CONFIRM author list CDDIS-21-2729RR

Dear colleagues,
Extensive revision experiments done by Anna M. Schläfli qualified her as a co-author on our paper. CDDis requires that all authors agree on the new author list (see below and attachment).

"Please request agreement from all authors including additions and deletions, these can be collected in the following way:

Email your co-authors with the change, and ask them to reply to your email confirming that they agree to these changes. Once you have collected these replies, please combine all of the co-authors' email responses in one document and upload this file to your submission."

PLEASE REPLY TO THIS MAIL AND LET ME KNOW IF YOU AGREE. *Thank you.*

!! The manuscript submission can only continue once all authors replied !!

All the best,

Mario

**From:** EA F federzoni.elena@gmail.com
**Subject:** Re: important: PLEASE CONFIRM author list CDDIS-21-2729RR
**Date:** 13 April 2022 at 02:06
**To:** John Yates jyates@scripps.edu
**Cc:** Jean-emmanuel Sarry jean-emmanuel.sarry@inserm.fr, mario.tschan@pathology.unibe.ch, kristina.seiler@pathology.unibe.ch, magali.humbert@yahoo.fr, petra.minder@gmx.ch, mimashimo@gmail.com, anna.schlaefli@pathology.unibe.ch, deborah.shan@pathology.unibe.ch, bich.vu@unifr.ch, James Moresco jmoresco@scripps.edu, martin.sadowski@pathology.unibe.ch, ramin.radpour@dbmr.unibe.ch, thomas.kaufmann@pki.unibe.ch, joern.dengjel@unifr.ch, betorbet@uw.edu

---

I agree - Kudos to Anna for her work!

---

Virus-free. www.avg.com

Il giorno mar 12 apr 2022 alle ore 13:16 John Yates <jyates@scripps.edu> ha scritto:

> I agree to the inclusion of Anna as a co-author.
>
> John
>
> **Ernest W. Hahn Professor**
>
> Departments of Molecular Medicine and Neurobiology
>
> 10550 North Torrey Pines Road, SR302B
>
> The Scripps Research Institute
>
> LaJolla, CA 92037
>
> Scripps Research
> Science Changing Life
>
> **UPS/FEDEX ADDRESS:**
>
> **Attn: John Yates**
> **Rm: SR302 - Yates Lab**
> 9365 Waples Street, Suite E
> San Diego, CA 92121
>
> http://fields.scripps.edu
>
> ***Please add Yogi Slivers to Correspondence for meeting/travel coordination***
>
> **Admin: Yogi Slivers**
>
> **yslivers@scripps.edu**
>
> **858-784-8863**
>
> **858-784-8883**
>
> This email may contain material that is confidential and/or proprietary to The Scripps Research Institute for the sole use of the intended recipient. Any review, reliance or distribution by others or forwarding without express permission is strictly prohibited. If you are not the intended recipient, please contact the sender and delete all copies.

**From:** Iris Mashimo mimashimo@gmail.com
**Subject:** Re: important: PLEASE CONFIRM author list CDDIS-21-2729RR
**Date:** 13 April 2022 at 09:21
**To:** mario.tschan@pathology.unibe.ch
**Cc:** kristina.seiler@pathology.unibe.ch, magali.humbert@yahoo.fr, petra.minder@gmx.ch, anna.schlaefli@pathology.unibe.ch, deborah.shan@pathology.unibe.ch, federzoni.elena@gmail.com, bich.vu@unifr.ch, jmoresco@scripps.edu, jyates@scripps.edu, martin.sadowski@pathology.unibe.ch, ramin.radpour@dbmr.unibe.ch, thomas.kaufmann@pki.unibe.ch, jean-emmanuel.sarry@inserm.fr, joern.dengjel@unifr.ch, betorbet@uw.edu

---

Hello all,

I strongly agree with including Anna M. Schläfli as an author.

Hope everyone is doing well!

Best regards,
Iris

On Wednesday, April 13, 2022, mario.tschan@pathology.unibe.ch <mario.tschan@pathology.unibe.ch> wrote:

> Dear colleagues,
> Extensive revision experiments done by Anna M. Schläfli qualified her as a co-author on our paper. CDDis requires that all authors agree on the new author list (see below and attachment).
>
> "Please request agreement from all authors including additions and deletions, these can be collected in the following way:
>
> Email your co-authors with the change, and ask them to reply to your email confirming that they agree to these changes. Once you have collected these replies, please combine all of the co-authors' email responses in one document and upload this file to your submission."
>
> PLEASE REPLY TO THIS MAIL AND LET ME KNOW IF YOU AGREE. *Thank you.*
>
> !! The manuscript submission can only continue once all authors replied !!
>
> All the best,
>
> Mario

--
Sent from Gmail Mobile

**From:** DENGJEL Joern joern.dengjel@unifr.ch
**Subject:** RE: important: PLEASE CONFIRM author list CDDIS-21-2729RR
**Date:** 12 April 2022 at 22:02
**To:** mario.tschan@pathology.unibe.ch

---

I agree.

---

**From:** mario.tschan@pathology.unibe.ch <mario.tschan@pathology.unibe.ch>
**Sent:** Tuesday, April 12, 2022 8:41 PM
**To:** kristina.seiler@pathology.unibe.ch; magali.humbert@yahoo.fr; petra.minder@gmx.ch; mimashimo@gmail.com; anna.schlaefli@pathology.unibe.ch; deborah.shan@pathology.unibe.ch; federzoni.elena@gmail.com; VU Bich <bich.vu@unifr.ch>; jmoresco@scripps.edu; jyates@scripps.edu; martin.sadowski@pathology.unibe.ch; ramin.radpour@dbmr.unibe.ch; thomas.kaufmann@pki.unibe.ch; jean-emmanuel.sarry@inserm.fr; DENGJEL Joern <joern.dengjel@unifr.ch>; mario.tschan@pathology.unibe.ch; betorbet@uw.edu
**Subject:** important: PLEASE CONFIRM author list CDDIS-21-2729RR

Dear colleagues,
Extensive revision experiments done by Anna M. Schläfli qualified her as a co-author on our paper. CDDis requires that all authors agree on the new author list (see below and attachment).

"Please request agreement from all authors including additions and deletions, these can be collected in the following way:

Email your co-authors with the change, and ask them to reply to your email confirming that they agree to these changes. Once you have collected these replies, please combine all of the co-authors' email responses in one document and upload this file to your submission."

PLEASE REPLY TO THIS MAIL AND LET ME KNOW IF YOU AGREE. *Thank you.*

!! The manuscript submission can only continue once all authors replied !!

All the best,

Mario

**From:** James Moresco jmoresco@scripps.edu
**Subject:** Re: important: PLEASE CONFIRM author list CDDIS-21-2729RR
**Date:** 13 April 2022 at 14:14
**To:** mario.tschan@pathology.unibe.ch

---

Dear Mario,
I agree to the inclusion of Anna M. Schläfli as a co-author.
Have a great day,

James Moresco, Ph.D.
Staff Scientist
Yates Laboratory
Department of Molecular Medicine
The Scripps Research Institute
10550 North Torrey Pines Road, SR-111
La Jolla, CA 92037
(858) 784-9148

Shipping address:
The Scripps Research Institute
Yates Lab SR-111
9365 Waples St., Suite E
San Diego, CA 92121

---

**From:** mario.tschan@pathology.unibe.ch <mario.tschan@pathology.unibe.ch>
**Sent:** Tuesday, April 12, 2022 11:40 AM
**To:** kristina.seiler@pathology.unibe.ch <kristina.seiler@pathology.unibe.ch>; magali.humbert@yahoo.fr <magali.humbert@yahoo.fr>; petra.minder@gmx.ch <petra.minder@gmx.ch>; mimashimo@gmail.com <mimashimo@gmail.com>; anna.schlaefli@pathology.unibe.ch <anna.schlaefli@pathology.unibe.ch>; deborah.shan@pathology.unibe.ch <deborah.shan@pathology.unibe.ch>; federzoni.elena@gmail.com <federzoni.elena@gmail.com>; bich.vu@unifr.ch <bich.vu@unifr.ch>; James Moresco <jmoresco@scripps.edu>; John Yates <jyates@scripps.edu>; martin.sadowski@pathology.unibe.ch <martin.sadowski@pathology.unibe.ch>; ramin.radpour@dbmr.unibe.ch <ramin.radpour@dbmr.unibe.ch>; thomas.kaufmann@pki.unibe.ch <thomas.kaufmann@pki.unibe.ch>; jean-emmanuel.sarry@inserm.fr <jean-emmanuel.sarry@inserm.fr>; joern.dengjel@unifr.ch <joern.dengjel@unifr.ch>; mario.tschan@pathology.unibe.ch <mario.tschan@pathology.unibe.ch>; betorbet@uw.edu <betorbet@uw.edu>
**Subject:** important: PLEASE CONFIRM author list CDDIS-21-2729RR

Dear colleagues,
Extensive revision experiments done by Anna M. Schläfli qualified her as a co-author on our paper. CDDis requires that all authors agree on the new author list (see below and attachment).

"Please request agreement from all authors including additions and deletions, these can be collected in the following way:

Email your co-authors with the change, and ask them to reply to your email confirming that they agree to these changes. Once you have collected these replies, please combine all of the co-authors' email responses in one document and upload this file to your submission."

PLEASE REPLY TO THIS MAIL AND LET ME KNOW IF YOU AGREE. *Thank you.*

!! The manuscript submission can only continue once all authors replied !!

All the best,

Mario

**From:** John Yates jyates@scripps.edu
**Subject:** Re: important: PLEASE CONFIRM author list CDDIS-21-2729RR
**Date:** 12 April 2022 at 21:16
**To:** Jean-emmanuel Sarry jean-emmanuel.sarry@inserm.fr, mario.tschan@pathology.unibe.ch
**Cc:** kristina.seiler@pathology.unibe.ch, magali.humbert@yahoo.fr, petra.minder@gmx.ch, mimashimo@gmail.com, anna.schlaefli@pathology.unibe.ch, deborah.shan@pathology.unibe.ch, federzoni.elena@gmail.com, bich.vu@unifr.ch, James Moresco jmoresco@scripps.edu, martin.sadowski@pathology.unibe.ch, ramin.radpour@dbmr.unibe.ch, thomas.kaufmann@pki.unibe.ch, joern.dengjel@unifr.ch, betorbet@uw.edu

---

I agree to the inclusion of Anna as a co-author.

John

**Ernest W. Hahn Professor**
Departments of Molecular Medicine and Neurobiology
10550 North Torrey Pines Road, SR302B
The Scripps Research Institute
LaJolla, CA 92037

Scripps Research
Science Changing Life

**UPS/FEDEX ADDRESS:**
**Attn: John Yates**
**Rm: SR302 - Yates Lab**
9365 Waples Street, Suite E
San Diego, CA 92121
http://fields.scripps.edu

***Please add Yogi Slivers to Correspondence for meeting/travel coordination***
**Admin: Yogi Slivers**
**yslivers@scripps.edu**
**858-784-8863**
**858-784-8883**

This email may contain material that is confidential and/or proprietary to The Scripps Research Institute for the sole use of the intended recipient. Any review, reliance or distribution by others or forwarding without express permission is strictly prohibited. If you are not the intended recipient, please contact the sender and delete all copies.

---

**From:** Jean-emmanuel Sarry <jean-emmanuel.sarry@inserm.fr>
**Date:** Tuesday, April 12, 2022 at 11:45 AM
**To:** mario.tschan@pathology.unibe.ch <mario.tschan@pathology.unibe.ch>
**Cc:** kristina.seiler@pathology.unibe.ch <kristina.seiler@pathology.unibe.ch>, magali.humbert@yahoo.fr <magali.humbert@yahoo.fr>, petra.minder@gmx.ch <petra.minder@gmx.ch>, mimashimo@gmail.com <mimashimo@gmail.com>, anna.schlaefli@pathology.unibe.ch <anna.schlaefli@pathology.unibe.ch>, deborah.shan@pathology.unibe.ch <deborah.shan@pathology.unibe.ch>, federzoni.elena@gmail.com <federzoni.elena@gmail.com>, bich.vu@unifr.ch <bich.vu@unifr.ch>, James Moresco <jmoresco@scripps.edu>, John Yates <jyates@scripps.edu>, martin.sadowski@pathology.unibe.ch <martin.sadowski@pathology.unibe.ch>, ramin.radpour@dbmr.unibe.ch

**From:** Jean-emmanuel Sarry jean-emmanuel.sarry@inserm.fr
**Subject:** Re: important: PLEASE CONFIRM author list CDDIS-21-2729RR
**Date:** 12 April 2022 at 19:44
**To:** mario.tschan@pathology.unibe.ch
**Cc:** kristina.seiler@pathology.unibe.ch, magali.humbert@yahoo.fr, petra.minder@gmx.ch, mimashimo@gmail.com, anna.schlaefli@pathology.unibe.ch, deborah.shan@pathology.unibe.ch, federzoni.elena@gmail.com, bich.vu@unifr.ch, jmoresco@scripps.edu, jyates@scripps.edu, martin.sadowski@pathology.unibe.ch, ramin.radpour@dbmr.unibe.ch, thomas.kaufmann@pki.unibe.ch, joern.dengjel@unifr.ch, betorbet@uw.edu

---

Dear Mario

Yes I totally agree to this change.

Have a wonderful day,

JE

--
Dream bigger, anything is possible!
Driving scientific discovery through collaboration


--------------------------------------------------
**Jean-Emmanuel Sarry**

Team Leader & Principal Investigator
Team "Oncometabolism and Drug Resistance in Acute Myeloid Leukemia"
Equipe Labellisée LIGUE 2018
Centre de Recherches en Cancérologie de Toulouse - CRCT
UMR1037 Inserm/UMR5071 Cnrs
Université Toulouse III-Paul Sabatier

2 avenue Hubert Curien
31037 TOULOUSE CEDEX 1 - France
https://www.crct-inserm.fr/18-j-e-sarry/
https://twitter.com/jeansarry

Phone: (33) 582 74 16 32
Mobil: (33) 630 220 850


Le 2022-04-12 20:40, mario.tschan@pathology.unibe.ch a écrit :

> Dear colleagues,
> Extensive revision experiments done by Anna M. Schläfli qualified her as a co-author on our paper. CDDis requires that all authors agree on the new author list (see below and attachment).
>
> "Please request agreement from all authors including additions and deletions, these can be collected in the following way:
>
> Email your co-authors with the change, and ask them to reply to your email confirming that they agree to these changes. Once you have collected these replies, please combine all of the co-authors' email responses in one document and upload this file to your submission."
>
> PLEASE REPLY TO THIS MAIL AND LET ME KNOW IF YOU AGREE. *Thank you.*
>
> !! The manuscript submission can only continue once all authors replied !!
>
> All the best,
>
> Mario

CDDIS-21-2729
RR_ma...t.docx

**From:** Kaufmann, Thomas (PKI) thomas.kaufmann@pki.unibe.ch
**Subject:** Re: important: PLEASE CONFIRM author list CDDIS-21-2729RR
**Date:** 13 April 2022 at 08:23
**To:** Tschan, Mario (PATHOLOGY) mario.tschan@pathology.unibe.ch

Dear Mario,

I agree to the inclusion of Anna M. Schläfli as co-author.

Kind regards,

Thomas

---
Thomas Kaufmann, PhD
Associate Professor
Institute of Pharmacology
Medical Faculty
University of Bern
Inselspital, INO-F, office 56.F-603
CH-3010 Bern, Switzerland

Phone: +41 31 632 32 89 (office)
+41 31 632 25 23 (lab)
ORCiD Nr: 0000-0001-9906-874X
Email: thomas.kaufmann@pki.unibe.ch
http://www.pki.unibe.ch/research/research_groups/index_eng.html

---
This email is confidential. If you are not the intended recipient, you must not disclose or use the information contained in it. If you have received this email in error, please notify us immediately by return email and delete the document.

---

**From:** "Tschan, Mario (PATHOLOGY)" <mario.tschan@pathology.unibe.ch>
**Date:** Tuesday, 12 April 2022 at 20:40
**To:** "Seiler, Kristina (PATHOLOGY)" <kristina.seiler@pathology.unibe.ch>, Magali Humbert <magali.humbert@yahoo.fr>, "petra.minder@gmx.ch" <petra.minder@gmx.ch>, "mimashimo@gmail.com" <mimashimo@gmail.com>, "Bill, Anna Magdalena (PATHOLOGY)" <anna.schlaefli@pathology.unibe.ch>, "Krauer, Deborah (PATHOLOGY)" <deborah.shan@pathology.unibe.ch>, EA F <federzoni.elena@gmail.com>, "bich.vu@unifr.ch" <bich.vu@unifr.ch>, "jmoresco@scripps.edu" <jmoresco@scripps.edu>, "jyates@scripps.edu" <jyates@scripps.edu>, "Sadowski, Martin (PATHOLOGY)" <martin.sadowski@pathology.unibe.ch>, "Radpour, Ramin (DBMR)" <ramin.radpour@dbmr.unibe.ch>, "Kaufmann, Thomas (PKI)" <thomas.kaufmann@pki.unibe.ch>, Jean-emmanuel Sarry <jean-emmanuel.sarry@inserm.fr>, Jörn Dengjel <joern.dengjel@unifr.ch>, "Tschan, Mario (PATHOLOGY)" <mario.tschan@pathology.unibe.ch>, Bruce E Torbett <betorbet@uw.edu>
**Subject:** important: PLEASE CONFIRM author list CDDIS-21-2729RR

Dear colleagues,
Extensive revision experiments done by Anna M. Schläfli qualified her as a co-author on our paper. CDDis requires that all authors agree on the new author list (see below and attachment).

"Please request agreement from all authors including additions and deletions, these can be collected in the following way:

**From:** Kristina Seiler kristina.l.seiler@gmail.com
**Subject:** Re: important: PLEASE CONFIRM author list CDDIS-21-2729RR
**Date:** 12 April 2022 at 20:22
**To:** mario.tschan@pathology.unibe.ch

Hi Mario

I absolutely agree to add Anna Schläfli to the author list!

Thanks very much,
Kristina

On Tue, Apr 12, 2022, 1:44 PM <mario.tschan@pathology.unibe.ch> wrote:

> **From:** "Tschan, Mario (PATHOLOGY)" <matschan@campus.unibe.ch>
> **Subject: important: PLEASE CONFIRM author list CDDIS-21-2729RR**
> **Date:** 12 April 2022 at 19:40:51 GMT+1
> **To:** "Seiler, Kristina (PATHOLOGY)" <kristina.seiler@pathology.unibe.ch>, Magali Humbert <magali.humbert@yahoo.fr>, petra.minder@gmx.ch, mimashimo@gmail.com, "Bill, Anna Magdalena (PATHOLOGY)" <anna.schlaefli@pathology.unibe.ch>, "Krauer, Deborah (PATHOLOGY)" <deborah.shan@pathology.unibe.ch>, EA F <federzoni.elena@gmail.com>, bich.vu@unifr.ch, jmoresco@scripps.edu, jyates@scripps.edu, "Sadowski, Martin (PATHOLOGY)" <martin.sadowski@pathology.unibe.ch>, "Radpour, Ramin (DBMR)" <ramin.radpour@dbmr.unibe.ch>, "Kaufmann, Thomas (PKI)" <thomas.kaufmann@pki.unibe.ch>, Jean-emmanuel Sarry <jean-emmanuel.sarry@inserm.fr>, Jörn Dengjel <joern.dengjel@unifr.ch>, "Mario P. Tschan" <mario.tschan@pathology.unibe.ch>, Bruce E Torbett <betorbet@uw.edu>
>
> Dear colleagues,
> Extensive revision experiments done by Anna M. Schläfli qualified her as a co-author on our paper. CDDis requires that all authors agree on the new author list (see below and attachment).
>
> "Please request agreement from all authors including additions and deletions, these can be collected in the following way:
>
> Email your co-authors with the change, and ask them to reply to your email confirming that they agree to these changes. Once you have collected these replies, please combine all of the co-authors' email responses in one document and upload this file to your submission."
>
> PLEASE REPLY TO THIS MAIL AND LET ME KNOW IF YOU AGREE. *Thank you.*
>
> !! The manuscript submission can only continue once all authors replied !!
>
> All the best,
>
> Mario

**From:** Magali Humbert magali.humbert@yahoo.fr
**Subject:** Re: important: PLEASE CONFIRM author list CDDIS-21-2729RR
**Date:** 12 April 2022 at 20:29
**To:** mario.tschan@pathology.unibe.ch, Bruce E Torbett betorbet@uw.edu
**Cc:** kristina.seiler@pathology.unibe.ch, petra.minder@gmx.ch, mimashimo@gmail.com, anna.schlaefli@pathology.unibe.ch, deborah.shan@pathology.unibe.ch, federzoni.elena@gmail.com, bich.vu@unifr.ch, Jean-emmanuel Sarry jean-emmanuel.sarry@inserm.fr, jmoresco@scripps.edu, jyates@scripps.edu, martin.sadowski@pathology.unibe.ch, ramin.radpour@dbmr.unibe.ch, thomas.kaufmann@pki.unibe.ch, joern.dengjel@unifr.ch

---

Dear Mario,

I totally agree with Anna added to the co-author list.

Best,

Magali
Le mardi 12 avril 2022, 20:58:44 UTC+2, Bruce E Torbett <betorbet@uw.edu> a écrit :

Hi Mario – I strongly support Anna M. Schläfli be included as an author on the resubmission.

Best,

Bruce

----------------------------------------------------------------------------------------

***Bruce E. Torbett, PhD, MSPH***

Professor of Pediatrics,
School of Medicine,

University of Washington

And Seattle Children's Research Institute (SCRI)

Associate Director, Center for Immunity and Immunotherapies @ SCRI

Institute for Stem Cell and Regenerative Medicine

Co-Director, HIV interaction in Viral Evolution (HIVE) Center

Email: betorbet@uw.edu

Phone: 206-884-1140

FAX: 206-987-7310

Cell: 760-518-6730

**From:** Sadowski, Martin (PATHOLOGY) martin.sadowski@pathology.unibe.ch
**Subject:** RE: important: PLEASE CONFIRM author list CDDIS-21-2729RR
**Date:** 13 April 2022 at 06:58
**To:** EA F federzoni.elena@gmail.com, John Yates jyates@scripps.edu
**Cc:** Jean-emmanuel Sarry jean-emmanuel.sarry@inserm.fr, Tschan, Mario (PATHOLOGY) mario.tschan@pathology.unibe.ch, kristina.seiler@pathology.unibe.ch, magali.humbert@yahoo.fr, petra.minder@gmx.ch, mimashimo@gmail.com, Bill, Anna Magdalena (PATHOLOGY) anna.schlaefli@pathology.unibe.ch, Krauer, Deborah (PATHOLOGY) deborah.shan@pathology.unibe.ch, bich.vu@unifr.ch, James Moresco jmoresco@scripps.edu, Radpour, Ramin (DBMR) ramin.radpour@dbmr.unibe.ch, Kaufmann, Thomas (PKI) thomas.kaufmann@pki.unibe.ch, joern.dengjel@unifr.ch, betorbet@uw.edu

---

Dear all,

I agree to the inclusion of Anna Schläfli as co-author.

Kind regards,
Martin

Martin Sadowski, PhD
University of Bern
Institute of Pathology
Murtenstrasse 31
CH-3008 Bern

Tel.: +41 31 632 4991
Mail: martin.sadowski@pathology.unibe.ch
http://www.pathology.unibe.ch

**From:** EA F <federzoni.elena@gmail.com>
**Sent:** Mittwoch, 13. April 2022 03:06
**To:** John Yates <jyates@scripps.edu>
**Cc:** Jean-emmanuel Sarry <jean-emmanuel.sarry@inserm.fr>; Tschan, Mario (PATHOLOGY) <mario.tschan@pathology.unibe.ch>; kristina.seiler@pathology.unibe.ch; magali.humbert@yahoo.fr; petra.minder@gmx.ch; mimashimo@gmail.com; Bill, Anna Magdalena (PATHOLOGY) <anna.schlaefli@pathology.unibe.ch>; Krauer, Deborah (PATHOLOGY) <deborah.shan@pathology.unibe.ch>; bich.vu@unifr.ch; James Moresco <jmoresco@scripps.edu>; Sadowski, Martin (PATHOLOGY) <martin.sadowski@pathology.unibe.ch>; Radpour, Ramin (DBMR) <ramin.radpour@dbmr.unibe.ch>; Kaufmann, Thomas (PKI) <thomas.kaufmann@pki.unibe.ch>; joern.dengjel@unifr.ch; betorbet@uw.edu
**Subject:** Re: important: PLEASE CONFIRM author list CDDIS-21-2729RR

I agree - Kudos to Anna for her work!

---

Virus-free. www.avg.com

Il giorno mar 12 apr 2022 alle ore 13:16 John Yates <jyates@scripps.edu> ha scritto:

> I agree to the inclusion of Anna as a co-author.

**From:** Petra Minder petra.minder@gmx.ch
**Subject:** Aw: important: PLEASE CONFIRM author list CDDIS-21-2729RR
**Date:** 13 April 2022 at 07:42
**To:** mario.tschan@pathology.unibe.ch
**Cc:** kristina.seiler@pathology.unibe.ch, magali.humbert@yahoo.fr, mimashimo@gmail.com, anna.schlaefli@pathology.unibe.ch, deborah.shan@pathology.unibe.ch, federzoni.elena@gmail.com, bich.vu@unifr.ch, jmoresco@scripps.edu, jyates@scripps.edu, martin.sadowski@pathology.unibe.ch, ramin.radpour@dbmr.unibe.ch, thomas.kaufmann@pki.unibe.ch, jean-emmanuel.sarry@inserm.fr, joern.dengjel@unifr.ch, mario.tschan@pathology.unibe.ch, betorbet@uw.edu

Dear all,

I agree to the inclusion of Anna M. Schläfli as co-author.

Kind regards,

Petra

Petra Minder
Friedhofplatz 1
4500 Solothurn
petra.minder@gmx.ch
+47 79 817 82 34

**Gesendet:** Dienstag, 12. April 2022 um 20:40 Uhr
**Von:** mario.tschan@pathology.unibe.ch
**An:** kristina.seiler@pathology.unibe.ch, magali.humbert@yahoo.fr, petra.minder@gmx.ch, mimashimo@gmail.com, anna.schlaefli@pathology.unibe.ch, deborah.shan@pathology.unibe.ch, federzoni.elena@gmail.com, bich.vu@unifr.ch, jmoresco@scripps.edu, jyates@scripps.edu, martin.sadowski@pathology.unibe.ch, ramin.radpour@dbmr.unibe.ch, thomas.kaufmann@pki.unibe.ch, jean-emmanuel.sarry@inserm.fr, joern.dengjel@unifr.ch, mario.tschan@pathology.unibe.ch, betorbet@uw.edu
**Betreff:** important: PLEASE CONFIRM author list CDDIS-21-2729RR

Dear colleagues,
Extensive revision experiments done by Anna M. Schläfli qualified her as a co-author on our paper. CDDis requires that all authors agree on the new author list (see below and attachment).

"Please request agreement from all authors including additions and deletions, these can be collected in the following way:

Email your co-authors with the change, and ask them to reply to your email confirming that they agree to these changes. Once you have collected these replies, please combine all of the co-authors' email responses in one document and upload this file to your submission."

PLEASE REPLY TO THIS MAIL AND LET ME KNOW IF YOU AGREE. *Thank you.*

!! The manuscript submission can only continue once all authors replied !!

All the best,

Mario

**From:** Radpour, Ramin **(DBMR)** ramin.radpour@dbmr.unibe.ch
**Subject:** Re: Urgent: PLEASE CONFIRM author list CDDIS-21-2729RR
**Date:** 13 April 2022 at 17:12
**To:** Tschan, Mario (PATHOLOGY) mario.tschan@pathology.unibe.ch

---

Dear Mario,
I agree on adding Anna Schläfli as a co-author. She did all revision experiments.

Best,
Ramin Radpour
-------------------------------------
Sent from my mobile. Apologies for the brevity and typos.

> On 13 Apr 2022, at 13:33, Tschan, Mario (PATHOLOGY) <mario.tschan@pathology.unibe.ch> wrote:
>
> Dear all,
> Sorry to be so persistent, but our paper is stuck until I got a short mail from each co-author saying that they agree on adding Anna Schläfli as a co-author. She did all revision experiments. Our competitor in the field is also about to publish on non-metabolic HK2 functions in AML cells, and it would be really sad not to be the first ones to publish non-metabolic HK functions in AML.
>
> THANKS  a lot for a short mail.
>
> Cheers,
> Mario
>
>> Begin forwarded message:
>>
>> **From:** "Tschan, Mario (PATHOLOGY)" <mario.tschan@pathology.unibe.ch>
>> **Subject: important: PLEASE CONFIRM author list CDDIS-21-2729RR**
>> **Date:** 12 April 2022 at 19:40:52 GMT+1
>> **To:** "Seiler, Kristina (PATHOLOGY)" <kristina.seiler@pathology.unibe.ch>, Magali Humbert <magali.humbert@yahoo.fr>, "petra.minder@gmx.ch" <petra.minder@gmx.ch>, "mimashimo@gmail.com" <mimashimo@gmail.com>, "Bill, Anna Magdalena (PATHOLOGY)" <anna.schlaefli@pathology.unibe.ch>, "Krauer, Deborah (PATHOLOGY)" <deborah.shan@pathology.unibe.ch>, EA F <federzoni.elena@gmail.com>, "bich.vu@unifr.ch" <bich.vu@unifr.ch>, "jmoresco@scripps.edu" <jmoresco@scripps.edu>, "jyates@scripps.edu" <jyates@scripps.edu>, "Sadowski, Martin (PATHOLOGY)" <martin.sadowski@pathology.unibe.ch>, "Radpour, Ramin (DBMR)" <ramin.radpour@dbmr.unibe.ch>, "Kaufmann, Thomas (PKI)" <thomas.kaufmann@pki.unibe.ch>, Jean-emmanuel Sarry <jean-emmanuel.sarry@inserm.fr>, Jörn Dengjel <joern.dengjel@unifr.ch>, "Tschan, Mario (PATHOLOGY)" <mario.tschan@pathology.unibe.ch>, Bruce E Torbett <betorbet@uw.edu>
>>
>> Dear colleagues,
>> Extensive revision experiments done by Anna M. Schläfli qualified her as a co-author on our paper. CDDis requires that all authors agree on the new author list (see below and attachment).
>>
>> "Please request agreement from all authors including additions and deletions, these can be collected in the following way:
>>
>> Email your co-authors with the change, and ask them to reply to your email confirming that they agree to these changes. Once you have collected these replies, please combine all of the co-authors' email responses in one document and upload this file to your submission."
>>
>> PLEASE REPLY TO THIS MAIL AND LET ME KNOW IF YOU AGREE. *Thank you.*
>>
>> !! The manuscript submission can only continue once all authors replied !!
>>
>> All the best,
>>
>> Mario

CDDIS-21-2729
RR_ma...t.docx

**From:** VU Bich bich.vu@unifr.ch
**Subject:** RE: Urgent: PLEASE CONFIRM author list CDDIS-21-2729RR
**Date:** April 14, 2022 at 12:52 PM
**To:** mario.tschan@pathology.unibe.ch

VB

Dear Mario,

I agree on adding Anna Schläfli as a co-author.

Best regards

Bich Vu

---

**De :** mario.tschan@pathology.unibe.ch <mario.tschan@pathology.unibe.ch>
**Envoyé :** mercredi, 13 avril 2022 13:33:10
**À :** jmoresco@scripps.edu; james.moresco@utsouthwestern.edu; VU Bich; ramin.radpour@dbmr.unibe.ch
**Objet :** Urgent: PLEASE CONFIRM author list CDDIS-21-2729RR

Dear all,
Sorry to be so persistent, but our paper is stuck until I got a short mail from each co-author saying that they agree on adding Anna Schläfli as a co-author. She did all revision experiments. Our competitor in the field is also about to publish on non-metabolic HK2 functions in AML cells, and it would be really sad not to be the first ones to publish non-metabolic HK functions in AML.

THANKS  a lot for a short mail.

Cheers,
Mario

> Begin forwarded message:
>
> **From:** "Tschan, Mario (PATHOLOGY)" <mario.tschan@pathology.unibe.ch>
> **Subject: important: PLEASE CONFIRM author list CDDIS-21-2729RR**
> **Date:** 12 April 2022 at 19:40:52 GMT+1
> **To:** "Seiler, Kristina (PATHOLOGY)" <kristina.seiler@pathology.unibe.ch>, Magali Humbert <magali.humbert@yahoo.fr>, "petra.minder@gmx.ch" <petra.minder@gmx.ch>, "mimashimo@gmail.com" <mimashimo@gmail.com>, "Bill, Anna Magdalena (PATHOLOGY)" <anna.schlaefli@pathology.unibe.ch>, "Krauer, Deborah (PATHOLOGY)" <deborah.shan@pathology.unibe.ch>, EA F <federzoni.elena@gmail.com>, "bich.vu@unifr.ch" <bich.vu@unifr.ch>, "jmoresco@scripps.edu" <jmoresco@scripps.edu>, "jyates@scripps.edu" <jyates@scripps.edu>, "Sadowski, Martin (PATHOLOGY)" <martin.sadowski@pathology.unibe.ch>, "Radpour, Ramin (DBMR)" <ramin.radpour@dbmr.unibe.ch>, "Kaufmann, Thomas (PKI)" <thomas.kaufmann@pki.unibe.ch>, Jean-emmanuel Sarry <jean-emmanuel.sarry@inserm.fr>, Jörn Dengjel <joern.dengjel@unifr.ch>, "Tschan, Mario (PATHOLOGY)" <mario.tschan@pathology.unibe.ch>, Bruce E Torbett <betorbet@uw.edu>
>
> Dear colleagues,
> Extensive revision experiments done by Anna M. Schläfli qualified her as a co-author on our paper. CDDis requires that all authors agree on the new author list (see below and attachment).
>
> "Please request agreement from all authors including additions and deletions, these can be collected in the following way:
>
> Email your co-authors with the change, and ask them to reply to your email confirming that they agree to these changes. Once you have collected these replies, please combine all of the co-authors' email responses in one document and upload this file to your submission."
>
> PLEASE REPLY TO THIS MAIL AND LET ME KNOW IF YOU AGREE. *Thank you.*
>
> !! The manuscript submission can only continue once all authors replied !!
>
> All the best,
>
> Mario
